# Supplementary material for: Development and Validation of the Adolescent Sexting Scale (A-SextS) with a Spanish Sample
Source: Int J Environ Res Public Health. 2020 Oct 31;17(21):8042. doi: 10.3390/ijerph17218042 (PMC7663141; doi:10.3390/ijerph17218042)
Supplement: Supplementary file 1 [file ijerph-17-08042-s001.zip › Appendix 2. Studies included in the review of measures.docx]

1. Alfaro González, M.; Vázquez Fernández, M.E.; Fierro Urturi, A.; Herrero Bregón, B.; Muñoz Moreno, M.F.; Rodríguez Molinero, L. Uso y riesgos de las tecnologías de la información y comunicación en adolescentes de 13-18 años. *Acta Pediatr. Española* **2015**, *73*, e126–e135.

2. Arias Cerón, M.; Buendía Eisman, L.; Fernández Palomares, F. Grooming, Cyberbullying and Sexting in Chile according of sex and school management or administrative dependency. *Rev. Chil. Pediatría* **2018**, *89*, 352–360, doi:10.4067/S0370-41062018005000201.

3. Baiden, F.; Amankwah, J.; Owusu, A. Sexting among high school students in a metropolis in Ghana: An exploratory and descriptive study. *J. Child. Media* **2020**, Advance online publication, doi:10.1080/17482798.2020.1719854.

4. Baumgartner, S.E.; Sumter, S.R.; Peter, J.; Valkenburg, P.M.; Livingstone, S. Does country context matter? Investigating the predictors of teen sexting across Europe. *Comput. Human Behav.* **2014**, *34*, 157–164, doi:10.1016/j.chb.2014.01.041.

5. Beckmeyer, J.J.; Herbenick, D.; Fu, T.C.; Dodge, B.; Reece, M.; Fortenberry, J.D. Characteristics of Adolescent Sexting: Results from the 2015 National Survey of Sexual Health and Behavior. *J. Sex Marital Ther.* **2019**, *45*, 767–780, doi:10.1080/0092623X.2019.1613463.

6. Bermeo, F.I. Factores de riesgo sociodemográficos, familiares y personales asociados al Sexting en estudiantes de dos instituciones educativas de la ciudad de Cuenca, [Unpublished Master’s Thesis]. Universidad de Cuenca, 2019.

7. Brinkley, D.Y.; Ackerman, R.A.; Ehrenreich, S.E.; Underwood, M.K. Sending and receiving text messages with sexual content: Relations with early sexual activity and borderline personality features in late adolescence. *Comput. Human Behav.* **2017**, *70*, 119–130, doi:10.1016/j.chb.2016.12.082.

8. Campbell, S.W.; Park, Y.J. Predictors of mobile sexting among teens: Toward a new explanatory framework. *Mob. Media Commun.* **2014**, *2*, 20–39, doi:10.1177/2050157913502645.

9. Chaudhary, P.; Peskin, M.; Temple, J.R.; Addy, R.C.; Baumler, E.; Ross, S. Sexting and Mental Health: A School-Based Longitudinal Study among Youth in Texas. *J. Appl. Res. Child.* **2017**, *8*, Article 11.

10. Choi, H.J.; Mori, C.; Van Ouytsel, J.; Madigan, S.; Temple, J.R. Adolescent Sexting Involvement Over 4 Years and Associations With Sexual Activity. *J. Adolesc. Heal.* **2019**, *65*, 738–744, doi:10.1016/j.jadohealth.2019.04.026.

11. Cleary, H.M.D.; Najdowski, C.J. Awareness of Sex Offender Registration Policies and Self-Reported Sexual Offending in a Community Sample of Adolescents. *Sex. Res. Soc. Policy* **2019**, Advance online publication, doi:10.1007/s13178-019-00410-3.

12. Communications, C. *Teen online & wireless safety survey: cyberbullying, sexting, and parental controls.*; 2009;

13. Dawson, A.E.; Wymbs, B.T.; Evans, S.W.; DuPaul, G.J. Exploring how adolescents with ADHD use and interact with technology. *J. Adolesc.* **2019**, *71*, 119–137, doi:10.1016/j.adolescence.2019.01.004.

14. De Graaf, H.; Verbeek, M.; Van den Borne, M.; Meijer, S. Offline and Online Sexual Risk Behavior among Youth in the Netherlands: Findings from ‘Sex under the Age of 25’. *Front. public Heal.* **2018**, *6*, 1–10, doi:10.3389/fpubh.2018.00072.

15. Dodaj, A.; Sesar, K.; Jerinić, S. A Prospective Study of High-School Adolescent Sexting Behavior and Psychological Distress. *J. Psychol. Interdiscip. Appl.* **2020**, *154*, 111–128, doi:10.1080/00223980.2019.1666788.

16. Dolev-Cohen, M.; Ricon, T. Demystifying sexting: Adolescent sexting and its associations with parenting styles and sense of parental social control in Israel. *Cyberpsychology* **2020**, *14*, Article 6, doi:10.5817/CP2020-1-6.

17. Fix, R.L.; Falligant, J.M.; Alexander, A.A.; Burkhart, B.R. Race and victim age matter: Sexual behaviors and experiences among confined African American and European American youth with sexual and nonsexual offenses. *Sex. Abus.* **2019**, *31*, 50–72, doi:10.1177/1079063217720926.

18. Frankel, A.S.; Bass, S.B.; Patterson, F.; Dai, T.; Brown, D. Sexting, Risk Behavior, and Mental Health in Adolescents: An Examination of 2015 Pennsylvania Youth Risk Behavior Survey Data. *J. Sch. Health* **2018**, *88*, 190–199, doi:10.1111/josh.12596.

19. Gámez-Guadix, M.; de Santisteban, P. “Sex Pics?”: Longitudinal Predictors of Sexting Among Adolescents. *J. Adolesc. Heal.* **2018**, *63*, 608–614, doi:10.1016/j.jadohealth.2018.05.032.

20. Gámez-Guadix, M.; Mateos-Pérez, E. Longitudinal and reciprocal relationships between sexting, online sexual solicitations, and cyberbullying among minors. *Comput. Human Behav.* **2019**, *94*, 70–76, doi:10.1016/j.chb.2019.01.004.

21. Gámez-Guadix, M.; de Santisteban, P.; Resett, S. Sexting among Spanish adolescents: Prevalence and personality profiles. *Psicothema* **2017**, *29*, 29–34, doi:10.7334/psicothema2016.222.

22. Garitaonandia, C.; Karrera, I.; Larranaga, N. Media convergence, risk and harm to children online. *Doxa Comun.* **2019**, 179–199, doi:10.31921/doxacom.n28a10.

23. Gerding, A. Adolescent sexting: An examination of the psychosocial contributions to the creation and sharing of sexual images, Publication No. AAI10628994 [Doctoral Thesis, University of Missouri - Columbia] ProQuest Dissertations and Theses Global., 2016.

24. Gewirtz-Meydan, A.; Mitchell, K.J.; Rothman, E.E. What do kids think about sexting? *Comput. Human Behav.* **2018**, *86*, 256–265, doi:10.1016/j.chb.2018.04.007.

25. Ghorashi, Z.; Loripoor, M.; Lotfipur-Rafsanjani, S.M. Mobile access and sexting prevalence in high school students in rafsanjan city, Iran in 2015. *Iran. J. Psychiatry Clin. Psychol.* **2019**, *24*, 416–425, doi:10.32598/ijpcp.24.4.416.

26. Gregg, D.; Somers, C.L.; Pernice, F.M.; Hillman, S.B.; Kernsmith, P. Sexting Rates and Predictors From an Urban Midwest High School. *J. Sch. Health* **2018**, *88*, 423–433, doi:10.1111/josh.12628.

27. Gutiérrez Gómez, I. Estudio estadístico que muestra la presencia de las conductas (ciberbullying, sexting y cibergrooming) a través de la red social facebook, en los alumnos de la Escuela Secundaria Técnica n°1 “Andrés Álvaro García”, [Bachelor’s Thesis, Universidad Autónoma del Estado de México] Repositorio Institucional de la Universidad Autónoma del Estado de México., 2019.

28. Hinduja, S.; Patchin, J.W. *Sexting. A brief guide for educators and parents*; Cyberbullying Research Center., 2010;

29. Houck, C.D.; Barker, D.; Rizzo, C.; Hancock, E.; Norton, A.; Brown, L.K. Sexting and sexual behavior in at-risk adolescents. *Pediatrics* **2014**, *133*, e276–e282, doi:10.1542/peds.2013-1157.

30. Kim, S.; Martin-Storey, A.; Drossos, A.; Barbosa, S.; Georgiades, K. Prevalence and Correlates of Sexting Behaviors in a Provincially Representative Sample of Adolescents. *Can. J. Psychiatry* **2019**, Advance Online Publication, doi:10.1177/0706743719895205.

31. Kopecky, K. Sexting among Czech Preadolescents and Adolescents. *New Educ. Rev.* **2012**, *28*, 39–48.

32. Kopecky, K. Cyberbullying and Sexting between Children and Adolescents - Comparative Study. In Proceedings of the LUMEN 2014 - From theory to Inquiry in Social Sciences; Sandu, A., Caras, A., Eds.; Elsevier Ltd: Romania, 2014; Vol. 149, pp. 467–471.

33. Kopecky, K. Sexting among Slovak Pubescents and Adolescent Children. In Proceedings of the Internacional Conference EPC-TKS 2015; Vasile, C., Singer, F.., Stan, E., Eds.; Elsevier Ltd: SARA BURGERHARTSTRAAT 25, PO BOX 211, 1000 AE AMSTERDAM, NETHERLANDS, 2015; Vol. 203, pp. 244–250.

34. Lenhart, A. *Teens and Sexting: How and why minor teens are sending sexually suggestive nude or nearly nude images via text messaging*; Washington, DC, 2009;

35. León Prieto, M.E.; Vargas Romero, J.C.; Guillén Torres, I.G. El nivel de incidencia del sexting en adolescentes de 1ero a 3ero de bachillerato general unificado de la unidad educativa particular ‘Santo Domingo de Guzmán’ en el periodo 2016-2017. *Rev. Electrónica Psicol. Iztacala* **2017**, *20*, 165–181.

36. Lippman, J.R.; Campbell, S.W. Damned if you do, damned if you don’t…if you’re a girl: Relational and normative contexts of adolescent sexting in the United States. *J. Child. Media* **2014**, *8*, 371–386, doi:10.1080/17482798.2014.923009.

37. Livingstone, S.; Görzig, A. When adolescents receive sexual messages on the internet: Explaining experiences of risk and harm. *Comput. Human Behav.* **2014**, *33*, 8–15, doi:10.1016/j.chb.2013.12.021.

38. Lucić, M.; Baćak, V.; Štulhofer, A. The role of peer networks in adolescent pornography use and sexting in Croatia. *J. Child. Media* **2020**, *14*, 110–127, doi:10.1080/17482798.2019.1637356.

39. Maas, M.; Bray, B.; Noll, J. A Latent Class Analysis of Online Sexual Experiences and Offline Sexual Behaviors Among Female Adolescents. *J. Res. Adolesc.* **2018**, *28*, 731–747, doi:10.1111/jora.12364.

40. Maheux, A.J.; Evans, R.; Widman, L.; Nesi, J.; Prinstein, M.J.; Choukas-Bradley, S. Popular peer norms and adolescent sexting behavior. *J. Adolesc.* **2020**, *78*, 62–66, doi:10.1016/j.adolescence.2019.12.002.

41. Marcum, C.D.; Higgins, G.E.; Ricketts, M.L. Sexting behaviors among adolescents in rural North Carolina: A theoretical examination of low self-control and deviant peer association. *Int. J. Cyber Criminol.* **2014**, *8*, 68–78.

42. Medina, M.O.; Verdugo, C.K. Autoconcepto y sexting en adolescentes de 15 a 18 años en la ciudad de Cuenca, (Publication No. TPSC;84) [Bachelor’s Thesis, Universidad de Cuenca - Ecuador] Respositorio Institutcional de la Universidad de Cuenca., 2018.

43. Mishna, F.; Cook, C.; Gadalla, T.; Daciuk, J.; Solomon, S. Cyber bullying behaviors among middle and high school students. *Am. J. Orthopsychiatry* **2010**, *80*, 362–374, doi:10.1111/j.1939-0025.2010.01040.x.

44. Mitchell, K.J.; Finkelhor, D.; Jones, L.M.; Wolak, J. Prevalence and characteristics of youth sexting: A national study. *Pediatrics* **2012**, *129*, 13–20, doi:10.1542/peds.2011-1730.

45. Molla-Esparza, C.; López-González, E.; Losilla, J.M. Sexting prevalence and socio-demographic correlates in Spanish secondary school students. *Sex. Res. Soc. Policy* **2020**, Advance online publication, doi:10.1007/s13178-020-00434-0.

46. Montiel, I.; Carbonell, E.; Pereda, N. Multiple online victimization of Spanish adolescents: Results from a community sample. *Child Abus. Negl.* **2016**, *52*, 123–134, doi:10.1016/j.chiabu.2015.12.005.

47. Naezer, M. From risky behaviour to sexy adventures: reconceptualising young people’s online sexual activities. *Cult. Heal. Sex.* **2018**, *20*, 715–729, doi:10.1080/13691058.2017.1372632.

48. Nielsen, S.; Paasonen, S.; Spisak, S. ‘Pervy role-play and such’: girls’ experiences of sexual messaging online. *Sex Educ.* **2015**, *15*, 472–485, doi:10.1080/14681811.2015.1048852.

49. O’Sullivan, L.F. Linking Online Sexual Activities to Health Outcomes Among Teens. In *Positive and negative outcomes of sexual behaviors*; Lefkowitz, E.S., Vasilenko, S.A., Eds.; New Directions for Child and Adolescent Development; Wiley Periodicals, Inc.: 989 MARKET STREET, SAN FRANCISCO, CA 94103-1741 USA, 2014; Vol. 144, pp. 37–51.

50. Patrick, K.; Heywood, W.; Pitts, M.K.; Mitchell, A. Demographic and behavioural correlates of six sexting behaviours among Australian secondary school students. *Sex. Health* **2015**, *12*, 480–487, doi:10.1071/SH15004.

51. Quesada, S.; Fernández-González, L.; Calvete, E. El sexteo (sexting) en la adolescencia: Frecuencia y asociación con la victimización de ciberacoso y violencia en el noviazgo. *Behav. Psychol.* **2018**, *26*, 225–242.

52. Rice, E.; Rhoades, H.; Winetrobe, H.; Sanchez, M.; Montoya, J.; Plant, A.; Kordic, T. Sexually Explicit Cell Phone Messaging Associated With Sexual Risk Among Adolescents. *Pediatrics* **2012**, *130*, 667–673, doi:10.1542/peds.2012-0021.

53. Rice, E.; Gibbs, J.; Winetrobe, H.; Rhoades, H.; Plant, A.; Montoya, J.; Kordic, T. Sexting and sexual behavior among middle school students. *Pediatrics* **2014**, *134*, e21–e28, doi:10.1542/peds.2013-2991.

54. Rice, E.; Craddock, J.; Hemler, M.; Rusow, J.; Plant, A.; Montoya, J.; Kordic, T. Associations between sexting behaviors and sexual behaviors among mobile phone-owning teens in Los Angeles. *Child Dev.* **2018**, *89*, 110–117, doi:10.1111/cdev.12837.

55. Ricketts, M.L.; Maloney, C.; Marcum, C.D.; Higgins, G.E. The effect of Internet related problems on the sexting behaviors of juveniles. *Am. J. Crim. Justice* **2015**, *40*, 270–284, doi:10.1007/s12103-014-9247-5.

56. Ševčíková, A.; Blinka, L.; Daneback, K. Sexting as a predictor of sexual behavior in a sample of Czech adolescents. *Eur. J. Dev. Psychol.* **2018**, *15*, 426–437, doi:10.1080/17405629.2017.1295842.

57. Ševčíková, A. Girls’ and boys’ experience with teen sexting in early and late adolescence. *J. Adolesc.* **2016**, *51*, 156–162, doi:http://dx.doi.org/10.1016/j.adolescence.2016.06.007.

58. Soriano Ayala, E.; Cala, V.C.; Bernal Bravo, C. Factores socioculturales y psicológicos en el sexting adolescente: Un estudio transcultural. *Rev. Educ.* **2019**, *384*, 175–197, doi:10.4438/1988-592X-RE-2019-384-407.

59. Stanley, N.; Barter, C.; Wood, M.; Aghtaie, N.; Larkins, C.; Lanau, A.; Överlien, C. Pornography, Sexual Coercion and Abuse and Sexting in Young People’s Intimate Relationships: A European Study. *J. Interpers. Violence* **2018**, *33*, 2919–2944, doi:10.1177/0886260516633204.

60. Steinberg, D.B.; Simon, V.A.; Victor, B.G.; Kernsmith, P.D.; Smith-Darden, J.P. Onset Trajectories of Sexting and Other Sexual Behaviors Across High School: A Longitudinal Growth Mixture Modeling Approach. *Arch. Sex. Behav.* **2019**, *48*, 2321–2331, doi:10.1007/s10508-019-1414-9.

61. Strassberg, D.S.; McKinnon, R.K.; Sustaita, M.A.; Rullo, J. Sexting by High School Students: An Exploratory and Descriptive Study. *Arch. Sex. Behav.* **2013**, *42*, 15–21, doi:10.1007/s10508-012-9969-8.

62. Strassberg, D.S.; Rullo, J.E.; Mackaronis, J.E. The sending and receiving of sexually explicit cell phone photos (‘Sexting’) while in high school: One college’s students’ retrospective reports. *Comput. Human Behav.* **2014**, *41*, 177–183, doi:10.1016/j.chb.2014.09.008.

63. Strassberg, D.S.; Cann, D.; Velarde, V. Sexting by High School Students. *Arch. Sex. Behav.* **2017**, *46*, 1667–1672, doi:10.1007/s10508-016-0926-9.

64. Titchen, K.E.; Maslyanskaya, S.; Silver, E.J.; Coupey, S.M. Sexting and Young Adolescents: Associations with Sexual Abuse and Intimate Partner Violence. *J. Pediatr. Adolesc. Gynecol.* **2019**, *32*, 481–486, doi:10.1016/j.jpag.2019.07.004.

65. Van Ouytsel, J.; Walrave, M.; Ponnet, K. Sexting within adolescents’ romantic relationships: How is it related to perceptions of love and verbal conflict? *Comput. Human Behav.* **2019**, *97*, 216–221, doi:10.1016/j.chb.2019.03.029.

66. Van Ouytsel, J.; Walrave, M.; Ponnet, K. An Exploratory Study of Sexting Behaviors Among Heterosexual and Sexual Minority Early Adolescents. *J. Adolesc. Heal.* **2019**, *65*, 621–626, doi:10.1016/j.jadohealth.2019.06.003.

67. Van Ouytsel, J.; Ponnet, K.; Walrave, M. The associations between adolescents’ consumption of pornography and music videos and their sexting behavior. *Cyberpsychol. Behav. Soc. Netw.* **2014**, *17*, 772–778, doi:10.1089/cyber.2014.0365.

68. Van Ouytsel, J.; Van Gool, E.; Ponnet, K.; Walrave, M. Brief report: The association between adolescents’ characteristics and engagement in sexting. *J. Adolesc.* **2014**, *37*, 1387–1391, doi:http://dx.doi.org/10.1016/j.adolescence.2014.10.004.

69. Vanden Abeele, M.; Roe, K.; Eggermont, S. An exploration of adolescents’ sexual contact and conduct risks through mobile phone use. *Communications* **2012**, *37*, 55–77, doi:10.1515/commun-2012-0003.

70. Velarde, V. The exchange of sexually explicit cell phone pictures (sexting) among high school students, (Publication No. 205929) [Bachelor’s Thesis, University of Utah - Utah] Digital Library of the University of Utah., 2014.

71. Villacampa, C. Sexting: prevalencia, características personales y conductuales y efectos en una muestra de adolescentes en España. *Rev. Gen. Derecho Penal* **2016**, *25*, 1–36.

72. Villanueva Blasco, V.J.; Serrano Bernal, S. Patrón de uso de internet y control parental de redes sociales como predictor de sexting en adolescentes: una perspectiva de género. *Rev. Psicol. y Educ.* **2019**, *14*, 16–26, doi:10.23923/rpye2019.01.168.

73. Wachs, S.; Wright, M.F.; Wolf, K.D. Psychological Correlates of Teen Sexting in three Countries - Direct and Indirect Associations between Self-control, Self-esteem, and Sexting. *Int. J. Dev. Sci.* **2017**, *11*, 109–120, doi:10.3233/DEV-160212.

74. Walrave, M.; Heirman, W.; Hallam, L. Under pressure to sext? Applying the theory of planned behaviour to adolescent sexting. *Behav. Inf. Technol.* **2014**, *33*, 86–98, doi:10.1080/0144929X.2013.837099.

75. West, J.H.; Lister, C.E.; Hall, P.C.; Crookston, B.T.; Snow, P.R.; Zvietcovich, M.E.; West, R.P. Sexting among peruvian adolescents. *BMC Public Health* **2014**, *14*, 811, doi:10.1186/1471-2458-14-811.

76. Wolfe, S.E.; Marcum, C.D.; Higgins, G.E.; Ricketts, M.L. Routine Cell Phone Activity and Exposure to Sext Messages: Extending the Generality of Routine Activity Theory and Exploring the Etiology of a Risky Teenage Behavior. *Crime Delinq.* **2013**, *62*, 614–644, doi:10.1177/0011128714541192.

77. Wood, M.; Barter, C.; Stanley, N.; Aghtaie, N.; Larkins, C. Images across Europe: The sending and receiving of sexual images and associations with interpersonal violence in young people’s relationships. *Child. Youth Serv. Rev.* **2015**, *59*, 149–160, doi:10.1016/j.childyouth.2015.11.005.

78. Woodward, V.H.; Evans, M.; Brooks, M. Social and Psychological Factors of Rural Youth Sexting: An Examination of Gender-Specific Models. *Deviant Behav.* **2017**, *38*, 461–476, doi:10.1080/01639625.2016.1197020.

79. Ybarra, M.L.; Mitchell, K.J. “Sexting” and Its Relation to Sexual Activity and Sexual Risk Behavior in a National Survey of Adolescents. *J. Adolesc. Heal.* **2014**, *55*, 757–764, doi:10.1016/j.jadohealth.2014.07.012.
