# Supplementary material for: Development and Validation of the Adolescent Sexting Scale (A-SextS) with a Spanish Sample
Source: Int J Environ Res Public Health. 2020 Oct 31;17(21):8042. doi: 10.3390/ijerph17218042 (PMC7663141; doi:10.3390/ijerph17218042)
Supplement: Supplementary file 1 [file ijerph-17-08042-s001.zip › Appendix 4. Refined version of A-SextS.docx]

**Preámbulo**

Se solicita su autorización para participar en el proyecto de investigación titulado: *Sexting: prevalencia, perfil de los participantes y operacionalización de la práctica.* Su objetivo es: *elaborar un instrumento de medida capaz de describir y analizar esta actividad de forma precisa*. Consiste en: *un cuestionario sencillo elaborado según criterios de calidad* *y adaptado a la población adolescente*. Los beneficios que se esperan de este trabajo: *se justifican por la necesidad de acercarnos a la realidad de los adolescentes en materia de relaciones sociales amorosas mediadas por la tecnología durante el transcurso de su etapa en educación secundaria*. La participación en este estudio es totalmente voluntaria, si usted no desea participar en el estudio, no habrá ninguna consecuencia negativa. En cualquier momento puede retirarse del estudio sin que ello tenga ninguna consecuencia. La respuesta es completamente anónima, por lo que no se dispondrá de ningún dato que pueda identificarle. En cualquier caso, la información se tratará de acuerdo a la Ley Orgánica 15/1999 de Protección de datos de Carácter Personal, de 6 de noviembre de 2019 (LOPD). Si tienen alguna pregunta sobre este proyecto de investigación, puede consultar en cualquier momento al investigador cuyo e-mail es: [________________](mailto:Cristian.Molla@uv.es)

Si usted responde a las cuestiones que se le proponen, se entiende de forma tácita que ha comprendido el objetivo del presente estudio, que ha podido preguntar y aclarar las dudas que se le hubieran planteado inicialmente y que acepta participar en el estudio. Los investigadores le agradecen su valiosa participación en el presente estudio.

**Instrucciones**

Este es un estudio sobre sexting. El sexting consiste en intercambiar mensajes de texto, audios, imágenes o videos sexys a través de internet con otra persona, y hacerlo con una intención amorosa o sexual. NO es 'sexting' enseñar todo o parte de tu cuerpo o de tu aspecto para conocer sólo la opinión de alguien, sin hacerlo con un objetivo amoroso o sexual

Se hace sexting con un objetivo amoroso o sexual, por ejemplo, para yo sentir placer, para que otro sienta placer, para llamar la atención sobre alguien, para demostrar deseo o interés sexual hacia alguien, como una prueba de amor para alguien, para saber si otras personas muestran deseo o interés sexual hacia mí, para describir un acto sexual que yo quiero hacer con alguien, para proponer tener sexo real o a través de internet, etc.

A continuación, encontrarás una serie de enunciados sobre sexting. Tu tarea consiste en rodear con un círculo (○) la frecuencia con la que has realizado cada una de estas acciones de sexting en el último mes (es decir, cuantas veces has hecho una acción concreta en los últimos 30 días). Junto con la acción que SI hayas realizado debes indicar con un círculo:

- Si enseñabas la cara *Toda o una parte* (
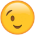
), *No la enseñabas* (
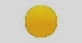
), o ambos.
- Si lo hiciste porque *Yo quería* (
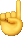
), *Sólo porque* *me lo habían pedido* (
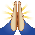
), o ambos.

*****

Recuerda que tu información será totalmente **CONFIDENCIAL**. Ni los maestros ni tus padres tendrán acceso a ella. Te pido que seas lo más **SINCERO/A** posible en tus respuestas.

*****

| \| Nunca = **0** \| **¡RECUERDA! 🡪 CON UN OBJETIVO AMOROSO O SEXUAL**  **/ ÚLTIMOS 30 DÍAS** \| \| \| \| --- \| --- \| --- \| --- \| \| Entre una y tres veces al mes = **1** \| **SÓLO** SI  has señalado 1, 2, 3 o 4  **puedes** rodear tantos EMOTICONOS como quieras \|  \| Enseñaba la cara (o una parte) \| \| \| Entre una y tres veces a la semana = **2** \| 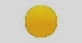 \| No enseñaba la cara \| \| \| Cada día o casi cada día = **3** \| 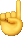 \| Porque yo quería \| \| \| Varias veces al día = **4** \| 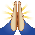 \| Porque me lo había pedido \| \| | Nunca |  | Entre 1 y 3 veces / mes | Entre 1 y 3 veces / semana | Cada día (o casi) | Varias veces al día |  | Enseñaba la cara | No enseñaba la cara | Porque yo quería 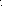 | Porque me lo había pedido |
| --- | --- | --- | --- | --- | --- | --- | --- | --- | --- | --- | --- | --- | --- | --- | --- | --- | --- | --- | --- | --- | --- | --- | --- | --- | --- | --- | --- | --- | --- | --- | --- | --- |
| **EJEMPLO** | **0** |  | **1** | **2** | **3** | **4** |  | 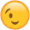 | 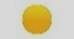 | 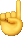 | 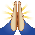 |
| He enviado una imagen o video de mi viaje a Italia **a mi novio/a** |  |  |  |  |  |  |  |  |  |  |  |
|  |  |  |  |  |  |  |  |  |  |  |  |
| He enviado un mensaje de texto sexy **a mi novio/a** | 0 |  | 1 | 2 | 3 | 4 |  |  |  | 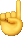 | 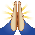 |
| He enviado un mensaje de texto sexy **a alguien que conozco en persona** | 0 |  | 1 | 2 | 3 | 4 |  |  |  | 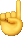 | 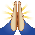 |
| He enviado un mensaje de texto sexy **a alguien que sólo conozco a través de Internet** | 0 |  | 1 | 2 | 3 | 4 |  |  |  | 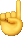 | 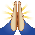 |
|  |  |  |  |  |  |  |  |  |  |  |  |
| He enviado un audio sexy (p. ej. mensaje de voz) **a mi novio/a** | 0 |  | 1 | 2 | 3 | 4 |  |  |  | 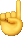 | 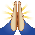 |
| He enviado un audio sexy (p. ej. mensaje de voz) **a alguien que conozco en persona** | 0 |  | 1 | 2 | 3 | 4 |  |  |  | 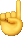 | 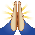 |
| He enviado un audio sexy (p. ej. mensaje de voz) **a alguien que sólo conozco a través de Internet** | 0 |  | 1 | 2 | 3 | 4 |  |  |  | 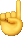 | 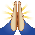 |
|  |  |  |  |  |  |  |  |  |  |  |  |
| He enviado una imagen o video de mis partes íntimas al desnudo **a mi novio/a** | 0 |  | 1 | 2 | 3 | 4 |  | 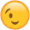 | 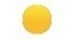 | 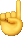 | 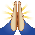 |
| He enviado una imagen o video de mis partes íntimas al desnudo **a alguien que conozco en persona** | 0 |  | 1 | 2 | 3 | 4 |  | 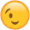 | 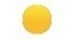 | 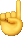 | 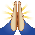 |
| He enviado una imagen o video de mis partes íntimas al desnudo **a alguien que sólo conozco a través de Internet** | 0 |  | 1 | 2 | 3 | 4 |  | 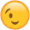 | 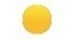 | 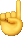 | 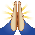 |
|  |  |  |  |  |  |  |  |  |  |  |  |
| He enviado una imagen o video de mis partes íntimas cubiertas con ropa interior **a mi novio/a** | 0 |  | 1 | 2 | 3 | 4 |  | 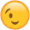 | 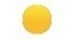 | 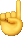 | 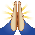 |
| He enviado una imagen o video de mis partes íntimas cubiertas con ropa interior **a alguien que conozco en persona** | 0 |  | 1 | 2 | 3 | 4 |  | 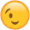 | 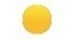 | 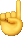 | 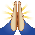 |
| He enviado una imagen o video de mis partes íntimas cubiertas con ropa interior **a alguien que sólo conozco a través de Internet** | 0 |  | 1 | 2 | 3 | 4 |  | 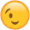 | 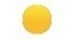 | 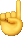 | 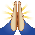 |
|  |  |  |  |  |  |  |  |  |  |  |  |
| He enviado una imagen o video de mí mismo vestido y en una postura sexy **a mi novio/a** | 0 |  | 1 | 2 | 3 | 4 |  | 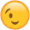 | 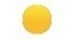 | 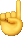 | 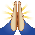 |
| He enviado una imagen o video de mí mismo vestido y en una postura sexy **a alguien que conozco en persona** | 0 |  | 1 | 2 | 3 | 4 |  | 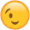 | 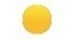 | 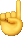 | 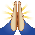 |
| He enviado una imagen o video de mí mismo vestido y en una postura sexy **a alguien que sólo conozco a través de Internet** | 0 |  | 1 | 2 | 3 | 4 |  | 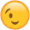 | 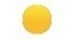 | 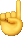 | 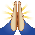 |
|  |  |  |  |  |  |  |  |  |  |  |  |
| He enviado una imagen o video sexy protagonizado por otros **a mi novio/a** | 0 |  | 1 | 2 | 3 | 4 |  |  |  | 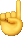 | 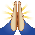 |
| He enviado una imagen o video sexy protagonizado por otros **a alguien que conozco en persona** | 0 |  | 1 | 2 | 3 | 4 |  |  |  | 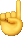 | 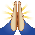 |
| He enviado una imagen o video sexy protagonizado por otros **a alguien que sólo conozco a través de Internet** | 0 |  | 1 | 2 | 3 | 4 |  |  |  | 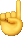 | 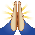 |
|  |  |  |  |  |  |  |  |  |  |  |  |
| He publicado un mensaje de texto (o comentario) sexy | 0 |  | 1 | 2 | 3 | 4 |  |  |  | 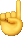 | 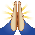 |
| He publicado una imagen o video de mis partes íntimas al desnudo | 0 |  | 1 | 2 | 3 | 4 |  | 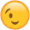 | 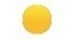 | 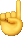 | 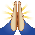 |
| He publicado una imagen o video de mis partes íntimas cubiertas con ropa interior | 0 |  | 1 | 2 | 3 | 4 |  | 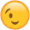 | 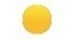 | 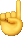 | 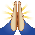 |
| He publicado una imagen o video de mí mismo vestido y en una postura sexy | 0 |  | 1 | 2 | 3 | 4 |  | 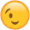 | 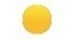 | 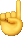 | 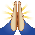 |
|  |  |  |  |  |  |  |  |  |  |  |  |
| He realizado *directos* (vídeos en directo) en el que mis partes íntimas estaban al desnudo | 0 |  | 1 | 2 | 3 | 4 |  | 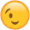 | 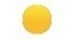 | 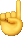 | 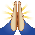 |
| He realizado *directos* (vídeos en directo) en el que mis partes íntimas estaban cubiertas con ropa interior | 0 |  | 1 | 2 | 3 | 4 |  | 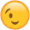 | 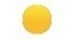 | 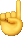 | 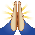 |
| He realizado *directos* (vídeos en directo) en el que estaba vestido y en una postura sexy | 0 |  | 1 | 2 | 3 | 4 |  | 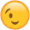 | 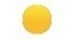 | 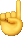 | 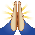 |
|  |  |  |  |  |  |  |  |  |  |  |  |
| He realizado llamadas de voz sexys (p.ej. llamadas telefónicas) **con mi novio/a** | 0 |  | 1 | 2 | 3 | 4 |  |  |  | 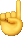 | 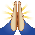 |
| He realizado llamadas de voz sexys (p.ej. llamadas telefónicas) **con alguien que conozco en persona** | 0 |  | 1 | 2 | 3 | 4 |  |  |  | 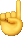 | 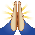 |
| He realizado llamadas de voz sexys (p.ej. llamadas telefónicas) **con alguien que sólo conozco a través de Internet** | 0 |  | 1 | 2 | 3 | 4 |  |  |  | 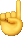 | 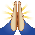 |
|  |  |  |  |  |  |  |  |  |  |  |  |

| \| Nunca = **0** \| **¡RECUERDA! 🡪 CON UN OBJETIVO AMOROSO O SEXUAL / ÚLTIMOS 30 DÍAS** \| \| \| \| --- \| --- \| --- \| --- \| \| Entre una y tres veces al mes = **1** \| **SÓLO** SI  has señalado 1, 2, 3 o 4  **puedes** rodear tantos EMOTICONOS como quieras \|  \| Enseñaba la cara (o una parte) \| \| \| Entre una y tres veces a la semana = **2** \| 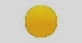 \| No enseñaba la cara \| \| \| Cada día o casi cada día = **3** \|  \| Porque yo quería \| \| \| Varias veces al día = **4** \|  \| Porque me lo había pedido \| \| | Nunca |  | Entre 1 y 3 veces / mes | Entre 1 y 3 veces / semana | Cada día (o casi) | Varias veces al día |  | Enseñaba la cara | No enseñaba la cara | Porque yo quería | Porque me lo había pedido |
| --- | --- | --- | --- | --- | --- | --- | --- | --- | --- | --- | --- | --- | --- | --- | --- | --- | --- | --- | --- | --- | --- | --- | --- | --- | --- | --- | --- | --- | --- | --- | --- | --- |
|  |  |  |  |  |  |  |  |  |  |  |  |
| **Con mi novio/a** he realizado vídeo-llamadas en las que mis partes íntimas estaban al desnudo | 0 |  | 1 | 2 | 3 | 4 |  |  |  |  |  |
| **Con alguien que conozco en persona** he realizado vídeo-llamadas en las que mis partes íntimas estaban al desnudo | 0 |  | 1 | 2 | 3 | 4 |  |  |  |  |  |
| **Con alguien que sólo conozco a través de Internet** he realizado vídeo-llamadas en las que mis partes íntimas estaban al desnudo | 0 |  | 1 | 2 | 3 | 4 |  |  |  |  |  |
|  |  |  |  |  |  |  |  |  |  |  |  |
| **Con mi novio/a** he realizado vídeo-llamadas en las que mis partes íntimas estaban cubiertas con ropa interior | 0 |  | 1 | 2 | 3 | 4 |  |  |  |  |  |
| **Con alguien que conozco en persona** he realizado vídeo-llamadas en las que mis partes íntimas estaban cubiertas con ropa interior | 0 |  | 1 | 2 | 3 | 4 |  |  |  |  |  |
| **Con alguien que sólo conozco a través de Internet** he realizado vídeo-llamadas en las que mis partes íntimas estaban cubiertas con ropa interior | 0 |  | 1 | 2 | 3 | 4 |  |  |  |  |  |
|  |  |  |  |  |  |  |  |  |  |  |  |
| **Con mi novio/a** he realizado vídeo-llamadas en las que estaba vestido y en una postura sexy | 0 |  | 1 | 2 | 3 | 4 |  |  |  |  |  |
| **Con alguien que conozco en persona** he realizado vídeo-llamadas en las que estaba vestido y en una postura sexy | 0 |  | 1 | 2 | 3 | 4 |  |  |  |  |  |
| **Con alguien que sólo conozco a través de Internet** he realizado vídeo-llamadas en las que estaba vestido y en una postura sexy | 0 |  | 1 | 2 | 3 | 4 |  |  |  |  |  |
|  |  |  |  |  |  |  |  |  |  |  |  |
| He pedido **a mi novio/a** que me envíe mensajes de texto sexys | 0 |  | 1 | 2 | 3 | 4 |  |  |  |  |  |
| He pedido **a alguien que conozco en persona** que me envíe mensajes de texto sexys | 0 |  | 1 | 2 | 3 | 4 |  |  |  |  |  |
| He pedido **a alguien que sólo conozco a través de Internet** que me envíe mensajes de texto sexys | 0 |  | 1 | 2 | 3 | 4 |  |  |  |  |  |
|  |  |  |  |  |  |  |  |  |  |  |  |
| He pedido **a mi novio/a** que me envíe audios sexys (p. ej. mensaje de voz) | 0 |  | 1 | 2 | 3 | 4 |  |  |  |  |  |
| He pedido **a alguien que conozco en persona** que me envíe audios sexys (p. ej. mensaje de voz) | 0 |  | 1 | 2 | 3 | 4 |  |  |  |  |  |
| He pedido **a alguien que sólo conozco a través de Internet** que me envíe audios sexys (p. ej. mensaje de voz) | 0 |  | 1 | 2 | 3 | 4 |  |  |  |  |  |
|  |  |  |  |  |  |  |  |  |  |  |  |
| He pedido **a mi novio/a** que me envíe imágenes o vídeos de sus partes íntimas al desnudo | 0 |  | 1 | 2 | 3 | 4 |  |  |  |  |  |
| He pedido **a alguien que conozco en persona** que me envíe imágenes o vídeos de sus partes íntimas al desnudo | 0 |  | 1 | 2 | 3 | 4 |  |  |  |  |  |
| He pedido **a alguien que sólo conozco a través de Internet** que me envíe imágenes o vídeos de sus partes íntimas al desnudo | 0 |  | 1 | 2 | 3 | 4 |  |  |  |  |  |
|  |  |  |  |  |  |  |  |  |  |  |  |
| He pedido **a mi novio/a** que me envíe imágenes o vídeos de sus partes íntimas cubiertas con ropa interior | 0 |  | 1 | 2 | 3 | 4 |  |  |  |  |  |
| He pedido **a alguien que conozco en persona** que me envíe imágenes o vídeos de sus partes íntimas cubiertas con ropa interior | 0 |  | 1 | 2 | 3 | 4 |  |  |  |  |  |
| He pedido **a alguien que sólo conozco a través de Internet** que me envíe imágenes o vídeos de sus partes íntimas cubiertas con ropa interior | 0 |  | 1 | 2 | 3 | 4 |  |  |  |  |  |
|  |  |  |  |  |  |  |  |  |  |  |  |
| He pedido **a mi novio/a** que me envíe imágenes o vídeos vestido y en una postura sexy | 0 |  | 1 | 2 | 3 | 4 |  |  |  |  |  |
| He pedido **a alguien que conozco en persona** que me envíe imágenes o vídeos vestido y en una postura sexy | 0 |  | 1 | 2 | 3 | 4 |  |  |  |  |  |
| He pedido **a alguien que sólo conozco a través de Internet** que me envíe imágenes o vídeos vestido y en una postura sexy | 0 |  | 1 | 2 | 3 | 4 |  |  |  |  |  |
|  |  |  |  |  |  |  |  |  |  |  |  |
| He pedido **a mi novio/a** hacer llamadas de voz sexys (p.ej. llamadas telefónicas) | 0 |  | 1 | 2 | 3 | 4 |  |  |  |  |  |
| He pedido **a alguien que conozco en persona** hacer llamadas de voz sexys (p.ej. llamadas telefónicas) | 0 |  | 1 | 2 | 3 | 4 |  |  |  |  |  |
| He pedido **a alguien que sólo conozco a través de Internet** hacer llamadas de voz sexys (p.ej. llamadas telefónicas) | 0 |  | 1 | 2 | 3 | 4 |  |  |  |  |  |
|  |  |  |  |  |  |  |  |  |  |  |  |
| He pedido **a mi novio/a** hacer vídeo-llamadas sexys | 0 |  | 1 | 2 | 3 | 4 |  |  |  |  |  |
| He pedido **a alguien que conozco en persona** hacer vídeo-llamadas sexys | 0 |  | 1 | 2 | 3 | 4 |  |  |  |  |  |
| He pedido **a alguien que sólo conozco a través de Internet** hacer vídeo-llamadas sexys | 0 |  | 1 | 2 | 3 | 4 |  |  |  |  |  |
|  |  |  |  |  |  |  |  |  |  |  |  |

**¡SIGUE RESPONDIENDO!**

| \| Nunca = **0** \| **¡RECUERDA! 🡪 CON UN OBJETIVO AMOROSO O SEXUAL / ÚLTIMOS 30 DÍAS** \| \| \| \| --- \| --- \| --- \| --- \| \| Entre una y tres veces al mes = **1** \| **SÓLO** SI  has señalado 1, 2, 3 o 4  **puedes** rodear tantos EMOTICONOS como quieras \|  \| Enseñaba la cara (o una parte) \| \| \| Entre una y tres veces a la semana = **2** \|  \| No enseñaba la cara \| \| \| Cada día o casi cada día = **3** \|  \| Porque yo quería \| \| \| Varias veces al día = **4** \|  \| Porque me lo había pedido \| \| | Nunca |  | Entre 1 y 3 veces / mes | Entre 1 y 3 veces / semana | Cada día (o casi) | Varias veces al día |  | Enseñaba la cara | No enseñaba la cara | Porque yo quería | Porque me lo había pedido |
| --- | --- | --- | --- | --- | --- | --- | --- | --- | --- | --- | --- | --- | --- | --- | --- | --- | --- | --- | --- | --- | --- | --- | --- | --- | --- | --- | --- | --- | --- | --- | --- | --- |
|  |  |  |  |  |  |  |  |  |  |  |  |
| **Mi novio/a** me ha pedido que le envíe contenidos sexys propios de algún tipo, pero no se los he querido enviar | 0 |  | 1 | 2 | 3 | 4 |  |  |  |  |  |
| **Alguien que conozco en persona** me ha pedido que le envíe contenidos sexys propios de algún tipo, pero no se los he querido enviar | 0 |  | 1 | 2 | 3 | 4 |  |  |  |  |  |
| **Alguien que sólo conozco a través de Internet** me ha pedido que le envíe contenidos sexys propios de algún tipo, pero no se los he querido enviar | 0 |  | 1 | 2 | 3 | 4 |  |  |  |  |  |
|  |  |  |  |  |  |  |  |  |  |  |  |
| He recibido contenidos sexys de algún tipo **de mi novio/a** | 0 |  | 1 | 2 | 3 | 4 |  |  |  |  |  |
| He recibido contenidos sexys de algún tipo **de alguien que conozco en persona** | 0 |  | 1 | 2 | 3 | 4 |  |  |  |  |  |
| He recibido contenidos sexys de algún tipo **de alguien que sólo conozco a través de Internet** | 0 |  | 1 | 2 | 3 | 4 |  |  |  |  |  |
|  |  |  |  |  |  |  |  |  |  |  |  |

**¡SIGUE RESPONDIENDO!**

| **1. Soy**  Chico  Chica  **2. Edad** | **3. Mis padres viven**  Juntos  Separados  Otro: ________ | **4. Orientación sexual** (Sentir atracción por)  Me gustan los chicos  Me gustan las chicas  Me gustan los chicos y las chicas  Otro: ____________  No lo sé / No me ha gustado nadie | | **5. El chico/a que me gusta/ba es o era …**  Más de tres años menor que yo  De uno a tres años menor que yo  De mi edad  De uno a tres años mayor que yo  Más de tres años mayor que yo  Todavía no me ha gustado nadie | **6. ¿Tienes novio o novia?**  En este momento SI  NO, pero antes sí he tenido  NO, todavía no he tenido novio/a | | **7. Tu novio/a es o era …**  Más de tres años menor que yo  De uno a tres años menor que yo  De mi edad  De uno a tres años mayor que yo  Más de tres años mayor que yo  Otro: ________________ |
| --- | --- | --- | --- | --- | --- | --- | --- |
| **8. ¿Cómo te informas sobre sexualidad?**  (Puedes señalar más de una casilla) | | | **9. Indica si:**  ¿Utilizas aplicaciones móviles específicas para ligar? (p.ej. Tinder)  **No** o  **Si**  ¿Ves porno?  **No** o  **Si ⏵ ¿Cuántas veces a la semana? ______** veces  ¿Te masturbas?  **No** o  **Si ⏵ ¿Veces por semana? ______** veces  ¿Has mantenido alguna relación sexual con penetración?  **No** o  **Si**  ¿Has mantenido alguna relación sexual sin realmente quererlo?  **No** o  **Si**  ¿Te consideras atractivo/a? Señala sólo una casilla.  **Nada  Poco  Bastante  Mucho** | | | **10. Cuando intercambias contenidos sexys, ¿Crees que…?**  (Puedes señalar más de una casilla)  Puede causar que hablen mal de mí o me rechacen Puede que enseñen mis mensajes/fotos/audios/vídeos sexys a otros Puedo tener problemas con la justicia en el futuro Puedo tener problemas para encontrar trabajo en el futuro Puedo sentirme mal en un futuro por haberlo hecho Puede que otra/s persona/s contacten conmigo sin yo hacer nada  Puede que otra/s persona/s me envíen contenidos sexys sin yo quererlo  Puede que utilice/n el contenido sexy para chantajearme, obligarme, …  Otros problemas: ______________________________________  Puede que no haya ningún problema o consecuencia | |
| Mis padres  Hermano/a  Otros familiares: ____________  Amigos/as  Novio/a  Escuela  Internet  Libros, revistas en papel etc.  Otros: ____________  No me informo / No contesto | | |  |  |  |  |  |

**Preamble**

Your consent is required to participate in the research project entitled: *“Sexting: Prevalence, Participant Profiles and Operationalization”*. Its goal is: *To develop a measuring instrument capable of accurately describing and analysing sexting*. It consists of: *A simple questionnaire prepared according to quality criteria and adapted for an adolescent population*. Expected outcomes: *A deeper understanding of adolescent amorous social relations mediated by technology during the period of secondary education*. Participation in this research project is entirely voluntary, and no negative consequences will result from your not participating. You can withdraw from the research project at any time without any consequence. Your answers will be treated anonymously, meaning that none of the data recorded will be identifiable with you personally. In any case, the data will be treated in accordance with Spanish Organic Law 15/1999 of the 6th of November 2019 on the Protection of Personal Data (Ley Orgánica 15/1999 de Protección de Datos de Carácter Personal, “LOPD”). If you have any questions about this research project, you can consult, at any time, the research contact whose e-mail is: ___________________

If you answer any of the questions proposed in this questionnaire, it shall be tacitly acknowledged that you have understood the goal of this research project, that you have been able to ask and clarify any doubts that you have had, and that you agree to your participation in the research project. The researchers thank you for your valuable participation in this research project.

**Instructions**

This is a research project on sexting. Sexting, as a term, is given to mean the exchange of sexy text messages, audios, images or videos over the internet with another person, and doing it with an amorous or sexual purpose. It is NOT given to mean showing all or part of your body or your appearance merely in order to know someone’s opinion, without doing so with an amorous or sexual purpose.

Therefore, you might partake in sexting, for example, in order to feel personal pleasure, to elicit someone else’s pleasure, to draw someone’s attention to you, to express sexual desire or interest towards someone, to provide proof of your amorous feelings towards someone, to seek confirmation of someone’s sexual desire or interest towards you, to describe a sexual act that you want to perform with someone, to propose having real or virtual sex, etc.

Below, you will find a series of statements about sexting. Your task is to circle (○) the frequency with which you have performed the indicated sexting action in the last month, i.e. how many times you have done it in the last 30 days. Next to the action that you have performed, indicate with a circle:

- If you have shown part or the whole of your face (), if you have not shown it (), or both.
- If you did it because you wanted to (), only because you had been asked to (), or both.

*****

Remember that the information you provide will be treated in **TOTAL CONFIDENTIALITY**. Neither your teachers nor your parents will have any access to it. Please be as **SINCERE** as possible in your answers.

*****

| \| Never = **0** \| **REMEMBER! 🡪 WITH AN AMOROUS OR SEXUAL PURPOSE - LAST 30 DAYS** \| \| \| \| --- \| --- \| --- \| --- \| \| Between one and three times a month = **1** \| **ONLY IF**  you have marked 1, 2, 3 or 4,  **you can** circle as many EMOTICONS as you want \|  \| Showing a part or all of my face \| \| Between one and three times a week = **2** \|  \| Not showing my face \| \| Every day or almost every day = **3** \|  \| Because I wanted to \| \| Several times a day = **4** \|  \| Because I was asked to \| | Never |  | Between 1 and 3 times a month | Between 1 and 3 times a week | Every day or almost every day | Several times a days |  | Showing my face | Not showing my face | Because I wanted to | Because I was asked to |
| --- | --- | --- | --- | --- | --- | --- | --- | --- | --- | --- | --- | --- | --- | --- | --- | --- | --- | --- | --- | --- | --- | --- | --- | --- | --- | --- | --- | --- |
| **EXAMPLE** | **0** |  | **1** | **2** | **3** | **4** |  |  |  |  |  |
| I have sent an image or video of my trip to Italy to **my boyfriend/girlfriend** |  |  |  |  |  |  |  |  |  |  |  |
|  |  |  |  |  |  |  |  |  |  |  |  |
| I have sent a sexy text message to **my boyfriend/girlfriend** | 0 |  | 1 | 2 | 3 | 4 |  |  |  |  |  |
| I have sent a sexy text message to **someone I know in person** | 0 |  | 1 | 2 | 3 | 4 |  |  |  |  |  |
| I have sent a sexy text message to **someone I only know on the internet** | 0 |  | 1 | 2 | 3 | 4 |  |  |  |  |  |
|  |  |  |  |  |  |  |  |  |  |  |  |
| I have sent a sexy audio (e.g. voice message) to **my boyfriend/girlfriend** | 0 |  | 1 | 2 | 3 | 4 |  |  |  |  |  |
| I have sent a sexy audio (e.g. voice message) to **someone I know in person** | 0 |  | 1 | 2 | 3 | 4 |  |  |  |  |  |
| I have sent a sexy audio (e.g. voice message) to **someone I only know on the internet** | 0 |  | 1 | 2 | 3 | 4 |  |  |  |  |  |
|  |  |  |  |  |  |  |  |  |  |  |  |
| I have sent an image or video of my naked private parts to **my boyfriend/girlfriend** | 0 |  | 1 | 2 | 3 | 4 |  |  |  |  |  |
| I have sent an image or video of my naked private parts to **someone I know in person** | 0 |  | 1 | 2 | 3 | 4 |  |  |  |  |  |
| I have sent an image or video of my naked private parts to **someone I only know on the internet** | 0 |  | 1 | 2 | 3 | 4 |  |  |  |  |  |
|  |  |  |  |  |  |  |  |  |  |  |  |
| I have sent an image or video of my private parts covered by underwear to **my boyfriend/girlfriend** | 0 |  | 1 | 2 | 3 | 4 |  |  |  |  |  |
| I have sent an image or video of my private parts covered by underwear to **someone I know in person** | 0 |  | 1 | 2 | 3 | 4 |  |  |  |  |  |
| I have sent an image or video of my private parts covered by underwear to **someone I only know on the internet** | 0 |  | 1 | 2 | 3 | 4 |  |  |  |  |  |
|  |  |  |  |  |  |  |  |  |  |  |  |
| I have sent an image or video of myself dressed and in a sexy pose to **my boyfriend/girlfriend** | 0 |  | 1 | 2 | 3 | 4 |  |  |  |  |  |
| I have sent an image or video of myself dressed and in a sexy pose to **someone I know in person** | 0 |  | 1 | 2 | 3 | 4 |  |  |  |  |  |
| I have sent an image or video of myself dressed and in a sexy pose to **someone I only know on the internet** | 0 |  | 1 | 2 | 3 | 4 |  |  |  |  |  |
|  |  |  |  |  |  |  |  |  |  |  |  |
| I have sent a sexy image or video featuring someone else to **my boyfriend/girlfriend** | 0 |  | 1 | 2 | 3 | 4 |  |  |  |  |  |
| I have sent a sexy image or video featuring someone else to **someone I know in person** | 0 |  | 1 | 2 | 3 | 4 |  |  |  |  |  |
| I have sent a sexy image or video featuring someone else to **someone I only know on the internet** | 0 |  | 1 | 2 | 3 | 4 |  |  |  |  |  |
|  |  |  |  |  |  |  |  |  |  |  |  |
| I have posted a sexy text message or comment | 0 |  | 1 | 2 | 3 | 4 |  |  |  |  |  |
| I have posted an image or video of my naked private parts | 0 |  | 1 | 2 | 3 | 4 |  |  |  |  |  |
| I have posted an image or video of my private parts covered by underwear | 0 |  | 1 | 2 | 3 | 4 |  |  |  |  |  |
| I have posted an image or video of myself dressed and in a sexy pose | 0 |  | 1 | 2 | 3 | 4 |  |  |  |  |  |
|  |  |  |  |  |  |  |  |  |  |  |  |
| I have made live-streamed video of my naked private parts | 0 |  | 1 | 2 | 3 | 4 |  |  |  |  |  |
| I have live-streamed video of my private parts covered by underwear | 0 |  | 1 | 2 | 3 | 4 |  |  |  |  |  |
| I have live-streamed video of myself dressed and in a sexy pose | 0 |  | 1 | 2 | 3 | 4 |  |  |  |  |  |
|  |  |  |  |  |  |  |  |  |  |  |  |
| I have made sexy voice calls (e.g. phone calls) with **my boyfriend/girlfriend** | 0 |  | 1 | 2 | 3 | 4 |  |  |  |  |  |
| I have made sexy voice calls (e.g. phone calls) with **someone I know in person** | 0 |  | 1 | 2 | 3 | 4 |  |  |  |  |  |
| I have made sexy voice calls (e.g. phone calls) with **someone I only know on the internet** | 0 |  | 1 | 2 | 3 | 4 |  |  |  |  |  |
|  |  |  |  |  |  |  |  |  |  |  |  |

| \| Never = **0** \| **REMEMBER! 🡪 WITH AN AMOROUS OR SEXUAL PURPOSE - LAST 30 DAYS** \| \| \| \| --- \| --- \| --- \| --- \| \| Between one and three times a month = **1** \| **ONLY IF**  you have marked 1, 2, 3 or 4,  **you can** circle as many EMOTICONS as you want \|  \| Showing a part or all of my face \| \| Between one and three times a week = **2** \|  \| Not showing my face \| \| Every day or almost every day = **3** \|  \| Because I wanted to \| \| Several times a day = **4** \|  \| Because I was asked to \| | Never |  | Between 1 and 3 times a month | Between 1 and 3 times a week | Every day or almost every day | Several times a days |  | Showing my face | Not showing my face | Because I wanted to | Because I was asked to |
| --- | --- | --- | --- | --- | --- | --- | --- | --- | --- | --- | --- | --- | --- | --- | --- | --- | --- | --- | --- | --- | --- | --- | --- | --- | --- | --- | --- | --- |
| I have made video calls with **my boyfriend/girlfriend** featuring my naked private parts | 0 |  | 1 | 2 | 3 | 4 |  |  |  |  |  |
| I have made video calls with **someone I know in person** featuring my naked private parts | 0 |  | 1 | 2 | 3 | 4 |  |  |  |  |  |
| I have made video calls with **someone I only know on the internet** featuring my naked private parts | 0 |  | 1 | 2 | 3 | 4 |  |  |  |  |  |
|  |  |  |  |  |  |  |  |  |  |  |  |
| I have made video calls with **my boyfriend/girlfriend** featuring my private parts covered by underwear | 0 |  | 1 | 2 | 3 | 4 |  |  |  |  |  |
| I have made video calls with **someone I know in person** featuring my private parts covered by underwear | 0 |  | 1 | 2 | 3 | 4 |  |  |  |  |  |
| I have made video calls with **someone I only know on the internet** featuring my private parts covered by underwear | 0 |  | 1 | 2 | 3 | 4 |  |  |  |  |  |
|  |  |  |  |  |  |  |  |  |  |  |  |
| I have made video calls with **my boyfriend/girlfriend** featuring myself dressed and in a sexy pose | 0 |  | 1 | 2 | 3 | 4 |  |  |  |  |  |
| I have made video calls with **someone I know in person** featuring myself dressed and in a sexy pose | 0 |  | 1 | 2 | 3 | 4 |  |  |  |  |  |
| I have made video calls with **someone I only know on the internet** featuring myself dressed and in a sexy pose | 0 |  | 1 | 2 | 3 | 4 |  |  |  |  |  |
|  |  |  |  |  |  |  |  |  |  |  |  |
| I have asked **my boyfriend/girlfriend** to send me sexy text messages | 0 |  | 1 | 2 | 3 | 4 |  |  |  |  |  |
| I have asked **someone I know in person** to send me sexy text messages | 0 |  | 1 | 2 | 3 | 4 |  |  |  |  |  |
| I have asked **someone I only know on the internet** to send me sexy text messages | 0 |  | 1 | 2 | 3 | 4 |  |  |  |  |  |
|  |  |  |  |  |  |  |  |  |  |  |  |
| I have asked **my boyfriend/girlfriend** to send me sexy audios (e.g. voice messages) | 0 |  | 1 | 2 | 3 | 4 |  |  |  |  |  |
| I have asked **someone I know in person** to send me sexy audios (e.g. voice messages) | 0 |  | 1 | 2 | 3 | 4 |  |  |  |  |  |
| I have asked **someone I only know on the internet** to send me sexy audios (e.g. voice messages) | 0 |  | 1 | 2 | 3 | 4 |  |  |  |  |  |
|  |  |  |  |  |  |  |  |  |  |  |  |
| I have asked **my boyfriend/girlfriend** to send me images or videos of their naked private parts | 0 |  | 1 | 2 | 3 | 4 |  |  |  |  |  |
| I have asked **someone I know in person** to send me images or videos of their naked private parts | 0 |  | 1 | 2 | 3 | 4 |  |  |  |  |  |
| I have asked **someone I only know on the internet** to send me images or videos of their naked private parts | 0 |  | 1 | 2 | 3 | 4 |  |  |  |  |  |
|  |  |  |  |  |  |  |  |  |  |  |  |
| I have asked **my boyfriend/girlfriend** to send me images or videos of their private parts covered by underwear | 0 |  | 1 | 2 | 3 | 4 |  |  |  |  |  |
| I have asked **someone I know in person** to send me pictures or videos of their private parts covered by underwear | 0 |  | 1 | 2 | 3 | 4 |  |  |  |  |  |
| I have asked **someone I only know on the internet** to send me images or videos of their private parts covered by underwear | 0 |  | 1 | 2 | 3 | 4 |  |  |  |  |  |
|  |  |  |  |  |  |  |  |  |  |  |  |
| I have asked **my boyfriend/girlfriend** to send me images or videos of them dressed and in a sexy pose | 0 |  | 1 | 2 | 3 | 4 |  |  |  |  |  |
| I have asked **someone I know in person** to send me images or videos of them dressed and in a sexy pose | 0 |  | 1 | 2 | 3 | 4 |  |  |  |  |  |
| I have asked **someone I only know on the internet** to send me images or videos of them dressed and in a sexy pose | 0 |  | 1 | 2 | 3 | 4 |  |  |  |  |  |
|  |  |  |  |  |  |  |  |  |  |  |  |
| I have asked **my boyfriend/girlfriend** to make a sexy voice call (e.g. phone call) with me | 0 |  | 1 | 2 | 3 | 4 |  |  |  |  |  |
| I have asked **someone I know in person** to make a sexy voice call (e.g. phone call) with me | 0 |  | 1 | 2 | 3 | 4 |  |  |  |  |  |
| I have asked someone I only know over the internet to make a sexy voice call (e.g. phone call) with me | 0 |  | 1 | 2 | 3 | 4 |  |  |  |  |  |
|  |  |  |  |  |  |  |  |  |  |  |  |
| I have asked **my boyfriend/girlfriend** to make a sexy video call with me | 0 |  | 1 | 2 | 3 | 4 |  |  |  |  |  |
| I have asked **someone I know in person** to make a sexy video call with me | 0 |  | 1 | 2 | 3 | 4 |  |  |  |  |  |
| I have asked **someone I only know on the internet** to make a sexy video call with me | 0 |  | 1 | 2 | 3 | 4 |  |  |  |  |  |
|  |  |  |  |  |  |  |  |  |  |  |  |

**KEEP ANSWERING!**

| \| Never = **0** \| **REMEMBER! 🡪 WITH AN AMOROUS OR SEXUAL PURPOSE - LAST 30 DAYS** \| \| \| \| --- \| --- \| --- \| --- \| \| Between one and three times a month = **1** \| **ONLY IF**  you have marked 1, 2, 3 or 4,  **you can** circle as many EMOTICONS as you want \|  \| Showing a part or all of my face \| \| Between one and three times a week = **2** \|  \| Not showing my face \| \| Every day or almost every day = **3** \|  \| Because I wanted to \| \| Several times a day = **4** \|  \| Because I was asked to \| | Never |  | Between 1 and 3 times a month | Between 1 and 3 times a week | Every day or almost every day | Several times a days |  | Showing my face | Not showing my face | Because I wanted to | Because I was asked to |
| --- | --- | --- | --- | --- | --- | --- | --- | --- | --- | --- | --- | --- | --- | --- | --- | --- | --- | --- | --- | --- | --- | --- | --- | --- | --- | --- | --- | --- |
|  |  |  |  |  |  |  |  |  |  |  |  |
| **My boyfriend/girlfriend** has asked me to send them sexy content of some kind, but I didn’t want to | 0 |  | 1 | 2 | 3 | 4 |  |  |  |  |  |
| **Someone I know in person** has asked me to send them some kind of sexy content, but I didn’t want to | 0 |  | 1 | 2 | 3 | 4 |  |  |  |  |  |
| **Someone I only know on the internet** has asked me to send them sexy content of some kind, but I didn’t want to | 0 |  | 1 | 2 | 3 | 4 |  |  |  |  |  |
|  |  |  |  |  |  |  |  |  |  |  |  |
| I have received sexy content of some kind from **my boyfriend/girlfriend** | 0 |  | 1 | 2 | 3 | 4 |  |  |  |  |  |
| I have received sexy content of some kind from **someone I know in person** | 0 |  | 1 | 2 | 3 | 4 |  |  |  |  |  |
| I have received sexy content of some kind from **someone I only know on the internet** | 0 |  | 1 | 2 | 3 | 4 |  |  |  |  |  |
|  |  |  |  |  |  |  |  |  |  |  |  |

**KEEP ANSWERING!**

| **1. I am**  Male  Female  **2. Age** | **3. My parents live**  Together  Are separated  Other: ________ | **4. Sexual orientation (attracted to)**  I like boys  I like girls  I like boys and girls  Other: ____________  I don’t know / There hasn’t been anyone I have liked | | **5. The boy/girl I like(d) is/was ...**  More than 3 years younger than me  1 to 3 years younger than me  My age  1 to 3 years older than me  More than 3 years older than me  There hasn’t been anyone I have liked | **6. Do you have boyfriend or girlfriend?**  Right now, YES  NO, but I had one before  NO, I have not had a boyfriend/girlfriend yet | | **7. Your boyfriend/girlfriend is/was**  More than 3 years younger than me  1 to 3 years younger than me  My age  1 to 3 years older than me  More than 3 years older than me  Other: ________________ |
| --- | --- | --- | --- | --- | --- | --- | --- |
| **8. How do you find out about sexuality?**  (You can check more than one box) | | | **9. Please indicate if:**  Do you use any specific mobile application for hooking up with people? (e.g. Tinder) No  or Yes  Do you watch porn? No or Yes  **⏵** How many times a week? ______ times  Do you masturbate? No or Yes  **⏵** Times per week? ______ times  Have you had penetrative sex? No or Yes  Have you had sexual relations without really wanting it? No  or Yes  Do you consider yourself attractive? Check only one box.  **Not at all A little  Quite a lot  A lot** | | | **10. When you exchange sexy content, do you think ... ?**  (You can check more than one box)  It can cause people to speak ill of me or reject me My sexy messages/photos/audios/videos might be shown to others I may have trouble with the law in the future I may have trouble finding a job in the future I may feel bad in the future for having done it  Other people may contact with me without me doing anything  Other people may send me sexy content without me wanting it  It may be used to blackmail me or force me to do something  It may lead to other problems: ________________________  It will not lead to any problems or consequences | |
| My parents  Brother/sister  Other family: ____________  Friends  Boyfriend/girlfriend  School  Internet  Books, paper magazines, etc.  Other: ____________  I am not informed / No answer | | |  |  |  |  |  |
