# Supplementary material for: Development and Validation of the Adolescent Sexting Scale (A-SextS) with a Spanish Sample
Source: Int J Environ Res Public Health. 2020 Oct 31;17(21):8042. doi: 10.3390/ijerph17218042 (PMC7663141; doi:10.3390/ijerph17218042)
Supplement: Supplementary file 1 [file ijerph-17-08042-s001.zip › en.Supplementary Materials.docx]

**Supplementary Material**

S1. *Transcription of the spoken instructions*

1. (DP1) Hello, for the next few minutes I will be your instructor. So please pay attention.
2. (DP2) This is a research project about sexting.
3. (DP3) Sexting is the exchange of sexy text messages, audio files, images or videos over the internet with another person, and with an amorous or sexual intention. Sexting is done with an amorous or sexual goal in mind, for example, in order to feel pleasure, to elicit someone else’s pleasure, to draw someone’s attention, to attract someone, to express sexual desire or interest towards someone, to provide proof of your amorous feelings towards someone, to propose some kind of sexual relation, to find out if another person likes you or has some kind of interest in you, and so on.
4. (DP4) The questionnaire you are going to fill out is VERY SIMPLE and has a FUN answer format. (…) The questionnaire asks whether you have exchanged sexy messages of any kind WITH YOUR BOYFRIEND OR GIRLFRIEND. (…) It also asks whether if you have exchanged sexy messages of any kind WITH SOMEONE YOU KNOW IN PERSON. (…) And it also asks whether you have exchanged sexy messages of any kind WITH SOMEONE YOU ONLY KNOW ON THE INTERNET.
5. (DP5) In particular, you are asked to indicate the FREQUENCY (…) with which you have done EACH of the stated acts (…) during the LAST MONTH.
6. (DP6) The frequency scale is this:

- ZERO indicates that you have NEVER done it.
- ONE indicates that you have done it between one and three times PER MONTH.
- TWO indicates that you have done it between one and three times PER WEEK.
- THREE indicates that you have done it every day or ALMOST EVERY DAY.
- FOUR indicates that you have done it SEVERAL TIMES A DAY.

Have you seen it? It is very easy to remember: never, between one and three times a month, between one and three times a week, every or almost every day, (…) or several times a day.

1. (DP7) If, for a statement, you have indicated 1, 2, 3 or 4, you should, as indicated by the arrow, CONTINUE TO THE RIGHT and circle THE CORRESPONDING EMOTICON!
2. (DP8) Let me explain the emoticons to you in detail.

The smiley emoticon (*) means that you DID SHOW your face in the content. If you DID NOT SHOW your face, circle the emoticon that does not have a face (*). If you sometimes showed your face and sometimes did not, you can circle BOTH emoticons.

The emoticon with an index finger pointing up (*) means that you did it BECAUSE YOU WANTED TO, while the emoticon with the pleading hands (*) indicates that you did it BECAUSE YOU WERE ASKED TO. If SOMETIMES you did it because you wanted to, and OTHER TIMES because you were asked to, you can circle BOTH emoticons. Or ... if you did it because you were asked to AND ALSO because you wanted to, you can again circle BOTH emoticons.

1. (DP9) Let’s see an example together.

The first statement says “I have sent a sexy image (…) to my boyfriend or girlfriend”. Since I have not done this during the last month, I’m going to circle ZERO and NOT touch any of the emoticons.

The second statement says “I have sent a sexy image (…) to someone I only know on the Internet”. Since I have done this EVERY DAY (OR ALMOST EVERY DAY), in the last month, I’m going to CIRCLE number THREE (…) AND go on to ANSWER WITH AN EMOTICON. Since I SHOWED MY FACE IN SOME PICTURES AND NOT IN OTHERS, I’m going to circle the emoticon of the smiley face and also the emoticon that has no face. Also, since I sent them BECAUSE I WAS ASKED TO, I’m going to circle the EMOTICON of the PLEADING HANDS. And so on, with EVERY ONE of the statements in the questionnaire.

1. (DP10) Ah! Remember that your participation in this research project is voluntary, and that your answers will be treated COMPLETELY ANONYMOUSLY. So do not write your NAME, SCHOOL YEAR, CLASS, SCHOOL, CITY or ANYTHING ELSE. No-one will know what you have answered.
2. (DP11) Please read the questionnaire slowly, and, if you have any questions, ask the person in charge. When you finish filling in the questionnaire, (…) fold it and leave it face down, (…) raise your hand, (…) and the person in charge will pass by with a box for you to put the questionnaire in. In this way, I can assure you your answers will be completely anonymous and confidential.

S2. *Additional notes on the administration of the discussion groups*

Prior to holding the discussion groups, seven primary school teachers (four men and three women, one of which was a therapeutic pedagogy teacher) were asked to provide a “cognitive debriefing” on the capacities of pre-adolescents, of 10 to 13 years old, in defining, distinguishing and discussing aspects about sexuality (e.g. sex-affective orientation, interpersonal relationships, body image, conditions that characterize each person’s sex, sexual practices, development of concrete and abstract thought, etc.). The information provided also helped the authors plan the discussion group guide and select topics that the youngsters were able to discuss easily.

The focus group guide was developed, pilot-tested with a small group of 4 tweens and teens, and revised before initiating the two main discussion groups.

To start, in the two main discussion groups, the purpose of the discussion group was explained to the adolescents, and some specific terms relating to sexting were provided. During the discussion, the adolescents contributed to face validity by providing us with an informative conceptual analysis of the actions that they thought were essential, common or uncommon, and those that we may have overlooked. They also suggested how to adjectivize the sexual media content exchanged in the practice of sexting. In addition, they commented on relevant aspects for assessing sexting. To end each discussion group, they talked about the meaning and clarity of aspects of the questionnaire, including its instructions, its organization, its questions, and the corresponding answer choices, in order to make sure that the questionnaire was clear for the final sample and would achieve the goal of the research project.

S3. *Discussion group guide*

**INSTRUCTIONS**

1. Sex (male or female 󠆽) and age.
2. Material: pen or pencil and a notebook.
3. Do not write your name or school year.
4. There are no right or wrong answers.
   1. Say exactly what you think. You won’t be judged in any way for it.
5. On the subject of discussion, I am not going to ask you if you have actually been involved in sexting or not. I’m not interested here. However, I do want to know how you think we should ask about sexting, whether those of us studying this phenomenon are asking the wrong questions.
6. For example, “Have you sent sexy or suggestive photos?”.
7. You will see me taking notes of interesting things that you may say.
8. These notes will be completely anonymous and confidential.

**ICT & SOCIAL MEDIA**

***Activity 1* Technological devices**

1. In your opinion, when someone talks about “technological devices”, what do they mean?

| 󠆽 Mobile phone | 󠆽 Computer | 󠆽 󠆽 Tablet or iPad | 󠆽 YouTube |
| --- | --- | --- | --- |
| 󠆽 Smart watch | 󠆽 WhatsApp | 󠆽 PlayStation | 󠆽 Instagram |
| 󠆽 Facebook | 󠆽 Headphones | 󠆽 Smart TV | 󠆽 Others: Which? ________________ |

1. Do you have your own mobile phone?
2. When can you say you have your own mobile phone?
3. To connect to the internet or social media, do you use devices that are yours or those of your parents?
4. How do you connect to the internet or social media from your mobile phone?
5. Do you us Wi-Fi or a mobile data connection? Do you do so at home or also in other places?
6. Have you ever brought your mobile phone to school?
7. Have you ever had your mobile phone confiscated or your internet connection taken away from you? Was that long ago?
8. Do your parents look at your mobile phone?
9. What do you use the internet and social media for?
10. Do you go online or on social media every day?
11. How many hours a day do you spend on the internet or on social media?
12. What social networks do you know about and use?
13. What do you use social media or do you think social media is for?
14. Are all the people in your social networks actual friends?

**SEXUAL ORIENTATION**

1. Do you know what it means to be asexual?
2. Do you know what it means to be homosexual?
3. Do you know what it means to be heterosexual?
4. Do you know what it means to be bisexual?
5. Do you know what it means to be pansexual?

**TYPES OF RELATIONSHIPS**

**Romantic relationships**

1. How do you know that you like a boy/girl? How do you realize?
2. If you like a boy/girl, does it mean you are dating?
   1. When is it that two people are dating?
   2. How do you call the phase when two people like each other, but still do not claim to be dating?
   3. How do you know when two people have stopped dating?
3. Has any boy or girl of your age had a boyfriend or girlfriend? What was that relationship like?
4. Have you ever had a boyfriend or girlfriend? The same age as you?
5. At your age, can a boy or girl have more than one boyfriend or girlfriend at the same time?
6. On a sexual level, what do you think you do with a boyfriend or girlfriend?

**Friendships**

1. So we already know what a boyfriend, girlfriend or partner is. Now, how would define a friend?
2. What characterizes a friendship?
3. What is the difference between a friendship and a romantic relationship?

**People you know in person**

1. Consider a boy or girl you have met in person, for example, at a summer camp, who you later have conversations with over the internet, for example, via WhatsApp. Would you consider that person a friend?
2. Could that person become a friend?
3. Do you have any friends like this?

**People you know on the internet**

1. Consider a boy or girl you have never met in person but only on the internet. Would you consider that person a friend?
2. Can a boy or girl you have met on the internet become a friend?
3. Do you have any friends like this?
4. Would you consider a person that you have met on the internet a stranger?

**People you do not know**

1. Is someone you do not know the same as a stranger?
2. Do you consider a person you have met or with whom you have been talking on the internet a stranger?
3. What characterizes a person who is a stranger for you?
4. Is someone you have never seen or spoken to before a stranger?
5. Is there any other kind of relationship you know of that I have not mentioned?

**WILLINGNESS & CONSENT**

1. When you send a friend a photo, whether sexy or not, do you ask them first if they want to receive it?
2. Has someone ever told you that they do not want to receive any more messages, for example, sexy or not sexy photos, or other kinds of content? Do you know or have you heard of that happening?
3. Have you ever told someone that you do not want to receive any more messages, for example, sexy or not sexy photos, or other kinds of content? Do you know or have you heard of that happening?
4. Have you ever felt pressured or coerced into sending someone a message, for example a sexy or not sexy photo, or other kind of content? Do you know or have you heard of that happening?

**SOCIAL MEDIA**

1. What can be done on social media?
2. Of all of these actions, what do you do the most?
3. Have you ever sent anyone sexy photos or videos featuring other people?

**SEXUALITY**

***Activity 2* Private parts and reproductive organs**

1. What do you consider to be the reproductive or sexual organs of the human body?

| 󠆽 Tongue | 󠆽 Breasts or nipples | 󠆽 Knees |
| --- | --- | --- |
| 󠆽 Penis | 󠆽 Hair | 󠆽 Vagina |
| 󠆽 Feet | 󠆽 Backside | 󠆽 Other: Which? |
| 󠆽 Lips | 󠆽 Navel | ______________ |

1. What do you consider as private parts of a boy’s body?

| 󠆽 Tongue | 󠆽 Breasts or nipples | 󠆽 Knees |
| --- | --- | --- |
| 󠆽 Penis/Vagina | 󠆽 Hair | 󠆽 Other: Which? |
| 󠆽 Feet | 󠆽 Backside | ______________ |
| 󠆽 Lips | 󠆽 Navel | ______________ |

1. What do you consider as private parts of a girl’s body?

| 󠆽 Tongue | 󠆽 Breasts or nipples | 󠆽 Knees |
| --- | --- | --- |
| 󠆽 Penis/Vagina | 󠆽 Hair | 󠆽 Other: Which? |
| 󠆽 Feet | 󠆽 Backside | ______________ |
| 󠆽 Lips | 󠆽 Navel | ______________ |

**GRAPHIC DESCRIPTION**

| **Column A (Nudes)** | **Column B (With Underwear)** | **Column C (Dressed and in sexy pose)** |
| --- | --- | --- |
| 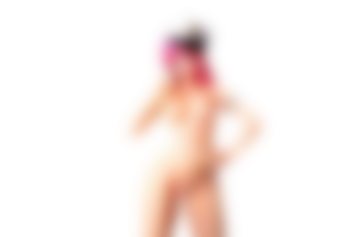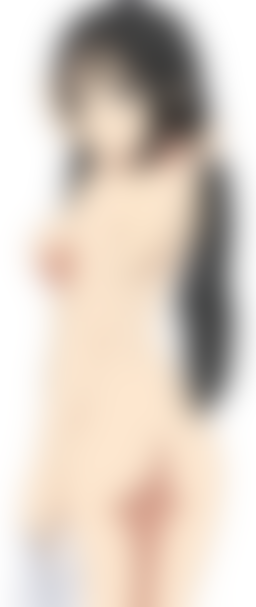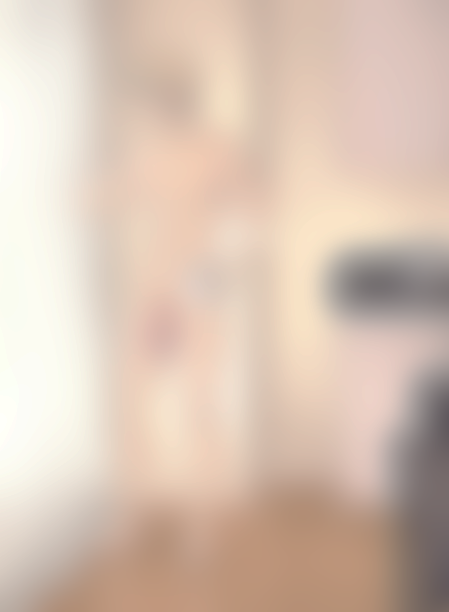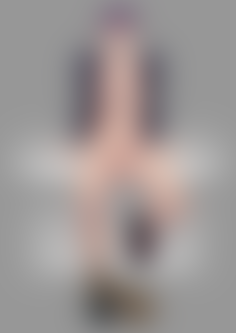 | 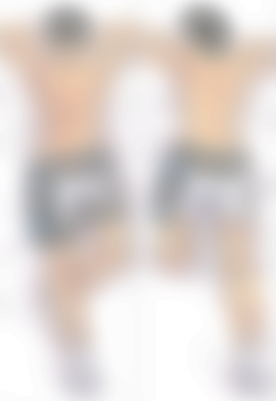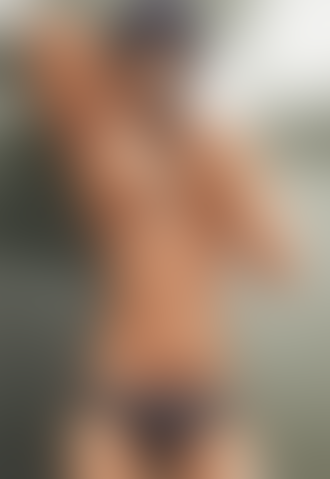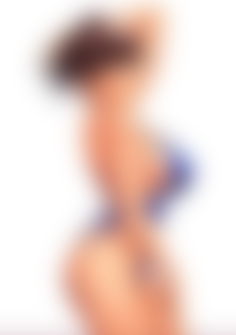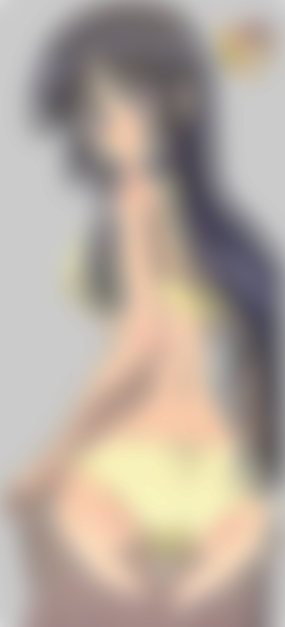 | 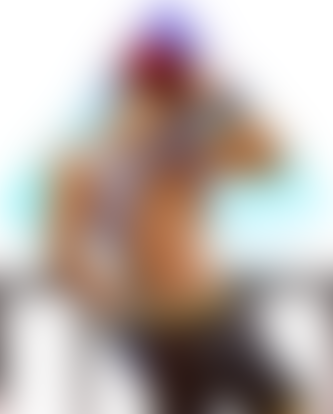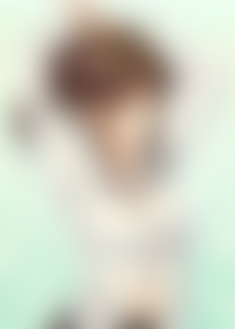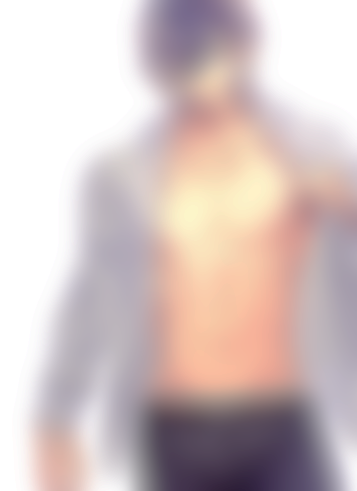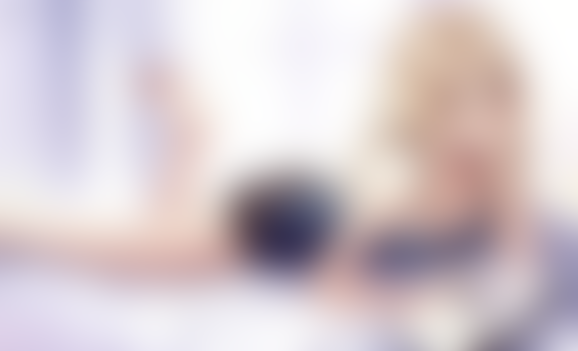 |

*** Activity 3 * Adjectivization of pictographic content**

1. What adjectives best describe the images in each column?
   1. First: Circle the 5 adjectives that best describe the images in each column.
   2. Second: Mark the adjective that best describes them with a 1 and that worst describes them with a 5.

|  | **COLUMN A** |  |  | **COLUMN B** |  |  | **COLUMN C** |
| --- | --- | --- | --- | --- | --- | --- | --- |
|  | Sexual activity |  |  | Sexual activity |  |  | Sexual activity |
|  | Almost naked |  |  | Almost naked |  |  | Almost naked |
|  | Covered by underwear |  |  | Covered by underwear |  |  | Covered by underwear |
|  | Nude |  |  | Nude |  |  | Nude |
|  | Erotic |  |  | Erotic |  |  | Erotic |
|  | Explicit |  |  | Explicit |  |  | Explicit |
|  | Inappropriate |  |  | Inappropriate |  |  | Inappropriate |
|  | Insinuating |  |  | Insinuating |  |  | Insinuating |
|  | Showing private parts |  |  | Showing private parts |  |  | Showing private parts |
|  | Showing a penis/vagina, breasts or nipples and/or backside |  |  | Showing a penis/vagina, breasts or nipples and/or backside |  |  | Showing a penis/vagina, breasts or nipples and/or backside |
|  | Partially naked |  |  | Partially naked |  |  | Partially naked |
|  | Provocative |  |  | Provocative |  |  | Provocative |
|  | Revealing |  |  | Revealing |  |  | Revealing |
|  | Seductive |  |  | Seductive |  |  | Seductive |
|  | Semi-naked |  |  | Semi-naked |  |  | Semi-naked |
|  | Sexy or sexual |  |  | Sexy or sexual |  |  | Sexy or sexual |
|  | Suggestive |  |  | Suggestive |  |  | Suggestive |
|  | Dressed and in a sexy pose |  |  | Dressed and in a sexy pose |  |  | Dressed and in a sexy pose |

1. Why did you choose these adjectives to describe the images in column A, B and C?

***Activity 4* The face**

1.
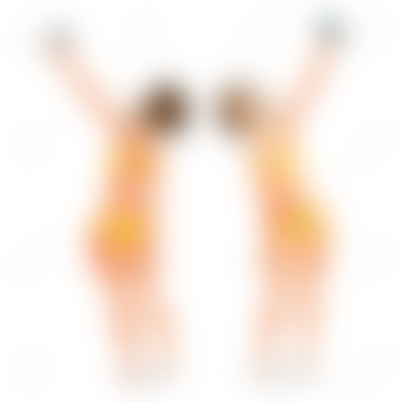

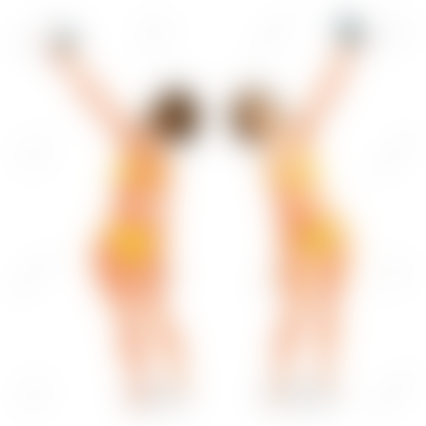
What differences can you see in these two images?

Imagen A

Imagen B

1. Is it important to ask whether the person’s face appears or not?

***Activity 5* Contents featuring other people**

1. Which statement is best understood? Mark with a 1 the one you think is best understood and with a 5 the one you think is the worst understood.

| 󠆽 I have sent a nude picture featuring another person.  󠆽 I have sent a nude picture of a stranger.  󠆽 I have sent a nude picture not of my own.  󠆽 I have sent a picture in which another person appears naked.  󠆽 I have sent a picture in which others appear naked. |
| --- |

*** Activity 6 * Discussion of the first draft of the questionnaire**

| - Your information will be totally CONFIDENTIAL, and neither your teachers nor your parents will have access to it, so I ask you to please be SINCERE in your answers. - Next, a set of actions are presented that have to do with the exchange of sexual content over the Internet with a love or sexual objective (a phenomenon called sexting). Next, a set of actions are presented that have to do with the exchange of sexual content over the Internet with a love or sexual objective (a phenomenon called sexting). Please indicate how often you have performed each of these actions in the last month (30 days). | | | | | | | | | | | | | | 0 = Never  1 = Between one and three times a month  2 = Between one and three times a week  3 = Every day or almost every day  4 = Several times a day  UP = I understand perfectly  NS = I am not sure if I understand perfectly  DU = I don’t understand | | | | |
| --- | --- | --- | --- | --- | --- | --- | --- | --- | --- | --- | --- | --- | --- | --- | --- | --- | --- | --- |
|  | | | | | | | | | | | | | |  | | | | |
| SENDING SEXY CONTENT OF YOURSELF: To feel pleasure, to make him/her feel pleasure, to express desire, to show sexual interest, to get attention, to find out whether other people have a sexual interest or desire for me, to describe a sexual act, to propose having real or virtual sex, to prove feelings, etc. | | | | | | | | | | | | | | | | | | |
| 1. I have sent my own text message of a sexual nature. | | | | | | | | | | | | | | | | UP | NS | DU |
| 1. I have sent my own audio message of a sexual nature. | | | | | | | | | | | | | | | | UP | NS | DU |
| 1. I have sent a picture or video of my nude private parts. | | | | | | | | | | | | | | | | UP | NS | DU |
| 1. I have sent an image or a video of my private parts covered by underwear. | | | | | | | | | | | | | | | | UP | NS | DU |
| 1. I have sent an image or video of myself dressed and in a sexy pose | | | | | | | | | | | | | | | | UP | NS | DU |
| 1. Indicate which of the previous actions you have carried out without being entirely sure or convinced. | | | | | | | | | | | | | | | | UP | NS | DU |
|  | No. 1 | | | No. 2 | | No. 3 | | No. 4 | | No. 5 | |  | | | |  |  |  |
| SEND SEXY CONTENT OF OTHERS: To feel pleasure, to make him/her feel pleasure, to express desire, to show sexual interest, to get attention, to find out whether other people have a sexual interest or desire for me, to describe a sexual act, to propose having real or virtual sex, to prove feelings, etc. | | | | | | | | | | | | | | | | | | |
| 1. I have sent a sexy text message written by another person and not by me. | | | | | | | | | | | | | | | | UP | NS | DU |
| 1. I have sent a sexy audio message of others that was not recorded by me. | | | | | | | | | | | | | | | | UP | NS | DU |
| 1. I have sent an image or video where other nudes appear. | | | | | | | | | | | | | | | | UP | NS | DU |
| 1. I have sent an image or video showing another person covered by underwear. | | | | | | | | | | | | | | | | UP | NS | DU |
| 1. I have sent an image or video where another person appears in a sexy pose. | | | | | | | | | | | | | | | | UP | NS | DU |
| 1. Indicate which of the previous actions you have carried out without being entirely sure or convinced. | | | | | | | | | | | | | | | | UP | NS | DU |
|  | No. 7 | | | No. 8 | | No. 9 | | No. 10 | | No. 11 | | No. 12 | | | |  |  |  |
| POSTING SEXY CONTENT OF YOURSELF: To feel pleasure, to make him/her feel pleasure, to express desire, to show sexual interest, to get attention, to find out whether other people have a sexual interest or desire for me, to describe a sexual act, to propose having real or virtual sex, to prove feelings, etc. | | | | | | | | | | | | | | | | | | |
| 1. I have posted my own sexy text message. | | | | | | | | | | | | | | | | UP | NS | DU |
| 1. I have posted an image or video of my nude private parts | | | | | | | | | | | | | | | | UP | NS | DU |
| 1. I have posted an image or video of my private parts covered by underwear | | | | | | | | | | | | | | | | UP | NS | DU |
| 1. I have posted an image or video of myself dressed and in a sexy pose. | | | | | | | | | | | | | | | | UP | NS | DU |
| 1. Indicate which of the previous actions you have carried out without being entirely sure or convinced. | | | | | | | | | | | | | | | | UP | NS | DU |
|  | No. 13 | | | No. 14 | | No. 15 | | No. 16 | | No. 17 | | | | | |  |  |  |
| LIVE BROADCASTING OR STREAMING YOUR OWN SEXY CONTENT: To feel pleasure, to make him/her feel pleasure, to express desire, to show sexual interest, to get attention, to find out whether other people have a sexual interest or desire for me, to describe a sexual act, to propose having real or virtual sex, to prove feelings, etc. | | | | | | | | | | | | | | | | | | |
| 1. I have broadcast my own sexy audio. | | | | | | | | | | | | | | | | UP | NS | DU |
| 1. I have broadcast a video or an image of my nude private parts. | | | | | | | | | | | | | | | | UP | NS | DU |
| 1. I have broadcast a video or image in which my private parts were covered by underwear. | | | | | | | | | | | | | | | | UP | NS | DU |
| 1. I have broadcast a video or image in which I was dressed and in a sexy pose. | | | | | | | | | | | | | | | | UP | NS | DU |
| 1. Indicate which of the previous actions you have carried out without being completely convinced or sure. | | | | | | | | | | | | | | | | UP | NS | DU |
|  | No. 18 | | | No. 19 | | No. 20 | | No. 21 | | No. 22 | | | | | |  |  |  |
| SEXY VOICE CALL: To feel pleasure, to make him/her feel pleasure, to express desire, to show sexual interest, to get attention, to find out whether other people have a sexual interest or desire for me, to describe a sexual act, to propose having real or virtual sex, to prove feelings, etc. | | | | | | | | | | | | | | | | | | |
| 1. I have made voice calls of a sexual nature. | | | | | | | | | | | | | | | | UP | NS | DU |
| 1. Check this box () if you have carried out the previous action (No. 23) without being completely convinced or sure. | | | | | | | | | | | | | | | | UP | NS | DU |
| SEXY VIDEO CALL: To feel pleasure, to make him/her feel pleasure, to express desire, to show sexual interest, to get attention, to find out whether other people have a sexual interest or desire for me, to describe a sexual act, to propose having real or virtual sex, to prove feelings, etc. | | | | | | | | | | | | | | | | | | |
| 1. I have made video calls in which my private parts were naked. | | | | | | | | | | | | | | | | UP | NS | DU |
| 1. I have made video calls in which my private parts were covered by underwear. | | | | | | | | | | | | | | | | UP | NS | DU |
| 1. I have made video calls in which I was dressed and in a sexy pose. | | | | | | | | | | | | | | | | UP | NS | DU |
| 1. Indicate which of the previous actions you have carried out without being entirely sure or convinced. | | | | | | | | | | | | | | | | UP | NS | DU |
|  | | No. 25 | | No. 26 | | No. 27 | | No. 28 | |  | | | | | |  |  |  |
| ASKING FOR SEXY CONTENT: To feel pleasure, to make him/her feel pleasure, to express desire, to show sexual interest, to get attention, to find out whether other people have a sexual interest or desire for me, to describe a sexual act, to propose having real or virtual sex, to prove feelings, etc. | | | | | | | | | | | | | | | | | | |
| 1. I have asked to be sent text messages of a sexual nature. | | | | | | | | | | | | | | | | UP | NS | DU |
| 1. I have asked to be sent audio messages of a sexual nature. | | | | | | | | | | | | | | | | UP | NS | DU |
| 1. I have asked to be sent images or videos of someone’s nude private parts. | | | | | | | | | | | | | | | | UP | NS | DU |
| 1. I have asked to be sent images or videos of someone’s private parts covered by underwear. | | | | | | | | | | | | | | | | UP | NS | DU |
| 1. I have asked to be sent pictures or videos of someone dressed and in a sexy pose. | | | | | | | | | | | | | | | | UP | NS | DU |
| 1. I have asked someone to live broadcast naked. | | | | | | | | | | | | | | | | UP | NS | DU |
| 1. I have asked someone to live broadcasts in their underwear. | | | | | | | | | | | | | | | | UP | NS | DU |
| 1. I have asked someone to do live broadcasts dressed and in a sexy pose. | | | | | | | | | | | | | | | | UP | NS | DU |
| 1. I have asked someone to make voice calls of a sexual nature. | | | | | | | | | | | | | | | | UP | NS | DU |
| 1. I have asked someone to make video calls in which my private parts were naked. | | | | | | | | | | | | | | | | UP | NS | DU |
| 1. I have asked someone to make video calls in which my private parts were covered by underwear. | | | | | | | | | | | | | | | | UP | NS | DU |
| 1. I have asked someone to make video calls in which I was dressed and in a sexy pose. | | | | | | | | | | | | | | | | UP | NS | DU |
| 1. Indicate which of the previous actions you have carried out without being entirely sure or convinced. | | | | | | | | | | | | | | | | UP | NS | DU |
| No. 29 | | | No. 30 | | No. 31 | | No. 32 | | No. 33 | | No. 34 | | No. 35 | | No. 36 |  |  |  |
| No. 37 | | | No. 38 | | No. 39 | | No. 40 | | No. 41 | |  | |  | |  |  |  |  |

S4. *Information on the databases, search strategy and descriptors used to conduct concurrent validity analysis*

| Databases | Published all years to |
| --- | --- |
| ERIC  Research field: in ANY FIELD | August  2020  (in all databases) |
| PsycINFO  Research field: in ANY FIELD |  |
| Pubmed  Research field: in ANY FIELD |  |
| Scopus  Research field: TITLE-ABS-KEY |  |
| Web of Science  Research field: in ANY FIELD |  |
| Grey literature |  |
| Google and Google Scholar  Where the words occur: anywhere in the publication | August  2005-2020 |
|  | The authors reviewed the first 250 results sorted by relevance |

*Note.* Descriptors were combined with Boolean operators.

*Note.* Exact words and related words were also used in the literature search.

(continued)

| Search terms | | | |
| --- | --- | --- | --- |
| Addressee 1 🡪 | Romantic partner  Boyfriend/Girlfriend  Partner  Committed partner  Romantic relationship  Lover  Life partner  Someone in relationship with | Addressee 2 🡪 | Friend  Acquaintance  Met in person  Know in person  Met only in person  Close friend  Somebody you know in person |
| Addressee 3 🡪 | Know online  Only knew online  Known through internet  Met only online  Had met online  Met on the Internet  Known from the internet  Online friend | Sending 🡪 | Sending  E-mailing  Messaging  Forward |
| Posting 🡪 | Posting  Uploaded  Placing on  Publicize  *Verb + on internet* | Live-broadcasting 🡪 | Live sex video  Video streaming  Video chat  Real time video  Real time video chat  Real time video application  Streaming  Web cam streaming  Online live streaming  Online sexual activity  Sexual self-exposure  Cybersex  In live video streaming  Web cam  Instagram story  Live-broadcasting  Broadcasting  In live video application  Live-action video |
| Voice call 🡪 | Voice call  Phone call  Telephone call | Video call 🡪 | Video call  Video record  Video file  Movies  Films  Videotape |
| Asking for 🡪 | Asking for a  Request a  Asked someone to  Asked your BF/GF to  Supplicate for a  Solicit  Make a petition  Require | Text messages 🡪 | Text  Written content  Reading material / content  Writings  Letters  Words |
| Image 🡪 | Image  Picture  Pic  Photo  Photograph | Video 🡪 | Video  Film  Picture  Movie  Video record |
| Audio 🡪 | Audio message  Audio  Audio record-ings  Voice record-ings  Audio record conversation  Audio clips  Audiotape | Nude 🡪 | Nude  Naked  Unclothed  Uncovered  Bare  Undressed  Disrobed  Garmentless |
| Underwear 🡪 | Lingerie  Undergarment  Skivvies  Underclothes  Underclothing  Underthings  Underpants  Panties  Boxer  Bra  G-string | Dressed 🡪 | Dressed  Clothed  Covered  Deck |
| Population 🡪 | Juvenile  Adolescent  Teenage / Teen  Junior  Young  Youth  Childish  Minors | Online sexual activities 🡪 | Online sexual activities  Internet sexual activities  Voluntary sexual exposure online  Sexy online self-presentations |

*Note.* In the literature search, different verb tenses and grammatical forms (e.g. singular, plural) of these terms were used.

S5. *Modular structure of the Adolescent Sexting Scale (A-SextS)*

| 1. Set of active experiences 2. In which there is an exchange of sexual representation media of varying formats and explicitness 3. Contextualized as an approximation to sexual-relational behaviour 4. Aimed at a more or less restricted audience 5. With an amorous or sexual purpose or responding to sexual objectives 6. In an approving framework (explicit or not) 7. During the last month | | | | |  | |
| --- | --- | --- | --- | --- | --- | --- |
|  |  |  |  |  |  | |
| **Timing** | **Experience** | **Appearance** | **Media** | **Sexual explicitness** | **Addressee** | **Channel / Audience** |
| Asynchronous ^Asy^ | Sending ^Se^ | Own ^Ow^ | Text ^Te^, Audio ^Au^ | Depending on the content ^Dc^ | Boy/Girl-fiend (Or prospective partner) ^Pa^  Boy/Girl I know personally (Friend/Ac.) ^Kp^  Boy/Girl I know through Internet (Online co.) ^Onl^ | Restricted audience ^Ra^ |
|  |  |  | Image/Video ^IV^ | Nude ^Nu^ |  |  |
|  |  |  |  | Underwear ^Un^ |  |  |
|  |  |  |  | Postural ^Pst^ |  |  |
|  |  | Non-own ^Now^ | Image/Video IV | Nude ^Nu^ | Boy/Girl-fiend (Or prospective partner) ^Pa^  Boy/Girl I know personally (Friend/Ac.) ^Kp^  Boy/Girl I know through Internet (Online co.) ^Onl^ | Restricted audience ^Ra^ |
|  |  |  |  | Underwear ^Un^ |  |  |
|  |  |  |  | Postural ^Pst^ |  |  |
| Synchronous ^Syn^ | Audio call ^Ac^ | - | Audio ^Au^ | Depending on the content ^Dc^ | Boy/Girl-fiend (Or prospective partner) ^Pa^  Boy/Girl I know personally (Friend/Ac.) ^Kp^  Boy/Girl I know through Internet (Online co.) ^Onl^ | Restricted audience ^Ra^ |
| Synchronous ^Syn^ | Video call ^Vc^ | - | Image/Video ^IV^ | Nude ^Nu^ | Boy/Girl-fiend (Or prospective partner) ^Pa^  Boy/Girl I know personally (Friend/Ac.) ^Kp^  Boy/Girl I know through Internet (Online co.) ^Onl^ | Restricted audience ^Ra^ |
|  |  |  |  | Underwear ^Un^ |  |  |
|  |  |  |  | Postural ^Pst^ |  |  |
| Asynchronous ^Asy^ | Posting ^Po^ | Own ^Ow^ | Text ^Te^ | Depending on the content ^Dc^ | Community or social network SN | Wide audience ^Wa^ |
|  |  |  | Image/Video ^IV^ | Nude ^Nu^ |  |  |
|  |  |  |  | Underwear ^Un^ |  |  |
|  |  |  |  | Postural ^Pst^ |  |  |
| Synchronous ^Syn^ | Broadcasting ^Brd^ | - | Image/Video ^IV^ | Nude ^Nu^ | Community or social network SN | Wide audience ^Wa^ |
|  |  |  |  | Underwear ^Un^ |  |  |
|  |  |  |  | Postural ^Pst^ |  |  |
| (continued) |  |  |  |  |  |  |

| Both ^Bth^ | Asking someone for ^Af^ | Send me ^Se^ | Text ^Te^, Audio ^Au^ | Depending on the content ^Dc^ | Boy/Girl-fiend (Or prospective partner) ^Pa^  Boy/Girl I know personally (Friend/Ac.) ^Kp^  Boy/Girl I know through Internet (Online co.) ^Onl^ |
| --- | --- | --- | --- | --- | --- |
|  |  |  | Image/Video ^IV^ | Nude ^Nu^ |  |
|  |  |  |  | Underwear ^Un^ |  |
|  |  |  |  | Postural ^Pst^ |  |
|  |  | Make audio calls ^Ac^ with me | Audio ^Au^ | Depending on the content ^Dc^ | Boy/Girl-fiend (Or prospective partner) ^Pa^  Boy/Girl I know personally (Friend/Ac.) ^Kp^  Boy/Girl I know through Internet (Online co.) ^Onl^ |
|  |  | Make video calls ^Vc^ with me | Image/Video ^IV^ | Depending on the content ^Dc^ | Boy/Girl-fiend (Or prospective partner) ^Pa^  Boy/Girl I know personally (Friend/Ac.) ^Kp^  Boy/Girl I know through Internet (Online co.) ^Onl^ |
| Asynchronous ^Asy^ | Asking me for, but I do not want to ^Den^ | Sending ^Se^ | General sexual media contents ^Smc^ | Generic ^Ge^  Depending on the content ^Dc^ | Boy/Girl-fiend (Or prospective partner) ^Pa^  Boy/Girl I know personally (Friend/Ac.) ^Kp^  Boy/Girl I know through Internet (Online co.) ^Onl^ |
| Asynchronous ^Asy^ | Receiving ^Rec^ | Receiving ^Rec^ | General sexual media contents ^Smc^ | Generic ^Ge^  Depending on the content ^Dc^ | Boy/Girl-fiend (Or prospective partner) ^Pa^  Boy/Girl I know personally (Friend/Ac.) ^Kp^  Boy/Girl I know through Internet (Online co.) ^Onl^ |

*Note.* Additional aspects included in the questionnaire were whether sexting was done on one’s own initiative or in response to a request (Vo) and whether the face of participants appeared in the pictographic sexts or not (Rec).

- Example of the modular structure of the Adolescent Sexting Scale.

For example, if a researcher wanted to study adolescent **active, primary and voluntary** sexting with a partner, 18 items would be selected.

| - Items | Experience | Media format | Explicitness | Addressee | Additional aspects |
| --- | --- | --- | --- | --- | --- |
| - Item 🡪 | Se | (Ow), Te | Dc | Pa | + Vo |
| - Item 🡪 | Se | (Ow), Au | Dc | Pa | + Vo |
| - Item 🡪 | Se | (Ow), IV | Nu | Pa | + Vo (+ Rec) |
| - Item 🡪 | Se | (Ow), IV | Un | Pa | + Vo (+ Rec) |
| - Item 🡪 | Se | (Ow), IV | Pst | Pa | + Vo (+ Rec) |
| - Item 🡪 | Ac | Au | Dc | Pa | + Vo |
| - Item 🡪 | Vc | IV | Nu | Pa | + Vo (+ Rec) |
| - Item 🡪 | Vc | IV | Un | Pa | + Vo (+ Rec) |
| - Item 🡪 | Vc | IV | Pst | Pa | + Vo (+ Rec) |
| - Item 🡪 | Af, Se | Te | Dc | Pa | + Vo |
| - Item 🡪 | Af, Se | Au | Dc | Pa | + Vo |
| - Item 🡪 | Af, Se | IV | Nu | Pa | + Vo |
| - Item 🡪 | Af, Se | IV | Un | Pa | + Vo |
| - Item 🡪 | Af, Se | IV | Pst | Pa | + Vo |
| - Item 🡪 | Af, Ac | Au | Dc | Pa | + Vo |
| - Item 🡪 | Af, Vc | IV | Dc | Pa | + Vo |
| - Item 🡪 | Den, Se | (Ow)Smc | Dc | Pa | + Vo |
|  |  | | | |  |

S6. *Analysis of sexting measures*

|  | A total of 79 studies sampling minors |
| --- | --- |
| Main transmission channels considered in primary sexting items | n=79* |
| Any ICT | 5 |
| Internet/Online | 17 |
| Social networks / Apps | 9 |
| Cell phone | 29 |
| E-mail | 9 |
| Messaging | 11 |
| Not defined / reported | 25 |
|  |  |
| Experiences considered in primary sexting items | n=79* |
| Sending (e.g. sending, texting, e-mailing, messaging, sharing with) | 69 |
| Receiving (e.g. receiving, seeing) | 50 |
| Forwarding (e.g. forwarding, sharing, distributing, disseminating, diffusing) | 16 |
| Posting (e.g. posting, sharing on, putting on, placing on) | 15 |
| Ask for (e.g. asking for, requesting, persisting, insist in) | 5 |
| Having a chat via web cam | 5 |
| Other experiences: Another 10 different experiences from 15 studies |  |
|  |  |
| Media content considered in primary sexting items | n=79* |
| Text | 36 |
| Images | 74 |
| Videos | 39 |
| Audios | 1 |
| Unclear | 2 |
|  |  |
| Adjectives used to characterize sexual media content in primary sexting items | n=79* |
| Sexy | 6 |
| Sexual | 30 |
| Only nude/naked | 5 |
| Only semi-nude/naked (e.g. semi-, nearly-, partially-, half-) | 1 |
| Nude/Naked or (…) | 27 |
| Semi-nude/naked or (…) | 23 |
| Sexually suggestive | 12 |
| Sexually explicit | 14 |
| Suggestive | 1 |
| Provocative | 4 |
| Underwear | 3 |
| Other adjectives: erotic, intimate, inappropriate, revealing, insinuating, sensitive, sexually toned, private, personal, seductive, etc. |  |
|  |  |
| Timeframe of the primary sexting items | n=79* |
| Lifetime or not defined | 45 |
| Last year | 17 |
| Last six months | 8 |
| Last month | 3 |
| Last three months | 1 |
| Last two months | 2 |
| Other timeframes: Another 4 different timeframes from 4 studies |  |
|  |  |
| Studies that set a purpose in primary sexting items (among items referring to sending or receiving sexting contents) (n=79) | |
| Yes | 2 |
| No | 76 |
| Unclear | 1 |
|  |  |
| Studies that set an addressee (for those sending) or sender (for those receiving) in either primary sexting items (n=78) | |
| Yes | 16 |
| No | 62 |
|  |  |
| Response type in primary sexting items | (n=79) |
| Likert frequency scale | 28 |
| Dichotomous | 24 |
| Single-answer polytomic | 3 |
| Monitoring conversation | 1 |
| Open-ended questions | 1 |
| Unclear | 22 |
|  |  |
| Evidence of validity and/or reliability in the study sample or in comparable samples (n=79) | |
| Yes | 15 |
| No | 57 |
| Unclear | 7 |
|  |  |
| Studies with at least one primary item of combined sexting (among those items referring to the experiences of sending and/or receiving). For example: sending or receiving, sending or posting, receiving or having shown. (n=78) | |
| Yes | 9 |
| No | 69 |
|  |  |
| Among those studies that consider the experience of sending | (n=69) |
| Nº of items used to asses this experience |  |
| 1 (Monoitem) | 34 |
| 2 | 4 |
| 3 | 7 |
| 4 | 2 |
| 5 | 0 |
| 6 | 1 |
| Monitoring conversations | 1 |
| Combined measure as a monoitem | 4 |
| Unclear | 16 |
|  |  |
| Among those studies that consider the action of receiving | (n=50) |
| Nº of items used to asses this experience |  |
| 1 (Monoitem) | 24 |
| 2 | 3 |
| 3 | 3 |
| Coding messages | 1 |
| Combined measure as a monoitem | 4 |
| Unclear | 15 |
|  |  |

* One study may report more than one category/level

S7. *Discussion group results*

Discussion Group 1 - (9:15)

| *Participants* | 4 boys and 6 girls | Age (11-12) | Duration: 90 minutes |
| --- | --- | --- | --- |
| 10 |  | Date: 10/01/2020 | Time: 9:15 am |

Discussion Group 2 - (11:30)

| *Participants* | 3 boys and 8 girls | Age (11-12) | Duration: 90 minutes |
| --- | --- | --- | --- |
| 11 |  | Date: 10/12/2019 | Time: 11:30 am |

*General information*

- Discussion group 1 took place in the multimedia room of the school.
- Discussion group 2 took place in the library of the school.

*Provided instructions*

- The researcher introduced himself.
- The researcher presented the topic, problem and goals of the research.
- The researcher informed the participants about the anonymous and voluntary nature of the activity.
- The researcher distributed the activity material to the participants.

*Session notes*

1. Internet, ICTs and social networks

Approximately half of the participants had their own mobile phone with some form of telecommunications service provider plan. The rest used their parents’ devices. All the participants agreed that their parents supervised the devices they used to a lesser or greater extent. Some of the most common parental measures were: knowing the passwords of the devices and/or social networks, sharing a profile on social media, monitoring conversations, using monitoring applications such as Family Link, and even having remote control of the device. Approximately a third of adolescents confused the term “technological devices”, including as such social networks or virtual platforms. The most appropriate term to refer to them was deemed “electronic devices”.

Internet access occurred mainly from within the family home and via a Wi-Fi connection. Not all participants had access to the internet every day, as this largely depended on their obligations and/or parental educational measures. On a normal or routine day, they would use the internet for between thirty and sixty minutes. The internet was used above all to view multimedia content, play games and/or search for information.

Participants were familiar with a good number of social networks (e.g. WhatsApp, Instagram, Facebook, Snapchat, TikTok, Twitter), though they tended to use the most popular among those of their age. They also knew about applications to search for romantic or sexual relationships (e.g. Tinder). Most of the adolescents had their own profile on at least one social network. They knew that it was possible to configure the privacy settings of social networks as public or private.

1. Types of relationships

The type of relationship they best knew how to define was friendship, characterized by daily and continued contact over time, trust, support and esteem. Dating relationships included the above characteristics in addition to attraction, both physical and because of the other’s way of being, and exclusivity. One of the participants commented that a certain sense of security, protection and affection were also expected to come from a boyfriend/girlfriend. Other elements that characterized a dating relationship were feeling comfortable with the other person and wanting to spend a lot of time with them. Regarding romantic relationships, students knew how to identify when they liked a person (e.g. he/she is handsome, he/she is nice and I want to always be with him/her, I get nervous when I see him/her, etc.).

The discussion group stated that there were already dating relationships between eleven and twelve-year-olds. Most of these relationships were between pupils in the same class or school, though they could also happen outside, such as with other participants in extracurricular activities. It was noted that the boyfriends or girlfriends were not always of the same age. A dating relationship was deemed to be consolidated when one person asked another to be his/her boyfriend/girlfriend and the latter answered affirmatively. The discussion group only noted kissing among possible sexual activities in dating relationships. The phase between friendship and established romantic relationships was termed “hooking up”. In conclusion, both friendship and dating were characterized by a common denominator: “personal interaction in real life” (textual words) and differentiated by a feeling of attraction or love.

Considered very different from the above types of relationships were those with people met via the internet and with people they had not met. According to almost all participants, people known exclusively via the internet could never become friends. One participant in particular did consider a young man met through an online video game team as a friend. The difference between an acquaintance on the internet and a stranger was the extent of knowledge or information possessed about that person, although the participants were fully aware that such information could be entirely made up or false. It was said that, “If you have never seen a stranger in person and never talked to him, you do not know anything about his life, and you have no intention doing so”.

1. Sexuality

They knew how to define “homosexuality”, “heterosexuality” and “bisexuality” as possible sexual orientations. Furthermore, these such terms were not considered pejorative by the participants. They believed that some students of their own age would not know the meaning of these terms. It was noted that such terms had been used in the content of teaching material during that particular school year.

They defined private parts as those parts of the body that are much more personal and that should be covered. They pointed out that there was a difference in considering the private parts of boys compared to those of girls. This difference lay in the consideration of breasts. Most of the students agreed that genitals and the backside, or bottom, were private parts of the bodies of boys. The private parts of the bodies of girls were considered to be genitals, breasts and the backside, or bottom.

1. Sexting

The participants did not know the term “sexting”, but they did know about the actions that define it. Most of the students admitted having received sexual content via the internet, mainly photographs and videos. Regarding consent, the adolescents pointed out that it was not usual to ask whether the other person wanted to receive any particular type of content. Notice was given only on some occasions, for example, with a message saying, “I’m going to send you a photo”. Nor was it considered common to tell the other person to stop sending content, except when they were copy-and-paste messages, such as Christmas greetings or other such types of messages. A couple of pupils said they had felt pressured to send content of some kind via the internet, especially via social networks and in group interactions, for example, via WhatsApp.

In sexting, understood as a type of dating dynamic, showing your face in sexy images or videos was not considered important. In this regard, the most important aspect for the participants was the showing of the private parts of the person featuring in the content, and the effect it could have on the receiver, in terms of emotion, excitement, and so on. Showing your face only became relevant when considering the consequences of any fraudulent use of this type of content. They proposed possible questions including the expressions “from the front or from the back”, “showing your whole body” and “seeing or knowing who you are”. According to them, the expressions “you are recognizable” or “seeing your face” would also be perfectly understood, but not the expression “you are identifiable”.

1. The scale

When evaluating the questionnaire on sexting, they considered that all potential actions were covered. However, they suggested some modifications (see below). One of the participants commented that, in reality, in no social network can a girl publish a nude photo or video because the platform itself censors such content. They recognized that you can publish images or videos with pixelated breasts, or with some element, including text, emojis or graphics, such as stars, covering the nipples. However, they admitted that, deep down, everyone knows that the person is naked in these types of images or videos. They also told the researcher about the existence of memes.

1. Item formulation
   - Describing nude content (Column A)
     - The most frequently mentioned terms to describe this type of content were “sexy” (n=17), “naked” (n=16), “inappropriate” (n=16), “private parts” (n=15) and “showing one’s penis/vagina, breasts and/or bottom” (n=15).
     - The terms that best (ranked 1st) described this type of content were “naked” (n=7) and “showing one’s penis/vagina, breasts and/or bottom (n=4).
   - Describing semi-nude contents (Column B)
     - The terms most frequently mentioned to describe this type of content were “covered by underwear” (n=16), “sexy” (n=16), “almost naked” (n=14) and “semi-naked” (n=12).
     - The terms that best (ranked 1st) described this type of content were “covered by underwear” (n=6), “almost naked” (n=5) and “semi-naked” (n=4).
   - Describing non-nude sexual content (Column C)
     - The terms most frequently mentioned to describe this type of content were “dressed and in a sexy pose” (n=21), “sexy” (n=16), “provocative” (n=14) and “erotic” (n=12).
     - The term that best (ranked 1st) described this type of content were “dressed and in a sexy pose” (n=13).
   - Referring to other people’s content
     - The term “featuring other people” (n=8) was that which the participants understood best (ranked 1st) when referring to content not created by themselves.
2. Understanding of the questionnaire items
   - - The instructions did not generate any confusion.
     - The frequency scale did not generate any confusion.
     - The items with the greatest difficulty of comprehension (<75% of positive evaluations) were: Item 1, Item 13, Item 2, Item 7, Item 18, Item 23, Item 34, Item 35, Item 36, Item 8, and Item 29. The participants explained why or what part of the item was not comprehensible.
       - - The groups suggested replacing the expression “of a sexual nature” with “sexy” in these items.
         - In items referring to text and audio messages, they proposed that examples be described right next to the item. Examples indicating the purpose or type of message. See the framed text in the template.
         - The groups suggested replacing “broadcasts” with “live broadcasts” or “live streaming”.
         - Eliminating the item about “broadcasting audio”: They would eliminate the live broadcasting of audio, as it is something considered unusual. What was considered most common were broadcasts in video, that is an audio-visual, format.
         - According to the discussion groups, “requesting someone to live broadcast”, whether naked, in underwear, and/or dressed and in a sexy pose is an unusual or infrequent action. They considered it “weird”.
         - The participants commented that asking for a video call with a love or sex interest can occur among adolescents, but they questioned the possibility of making such a request detailing the desired sexual explicitness of the call, such as naked, in underwear, and/or dressed and in a sexy pose. They considered that the above aspects would rather be broached in the course of a video call.
         - Other observations: In general, the adolescents had some difficulties understanding long or complex items.
